# Heusler alloys for spintronic devices: review on recent development and future perspectives

**DOI:** 10.1080/14686996.2020.1812364

**Published:** 2021-03-29

**Authors:** Kelvin Elphick, William Frost, Marjan Samiepour, Takahide Kubota, Koki Takanashi, Hiroaki Sukegawa, Seiji Mitani, Atsufumi Hirohata

**Affiliations:** a Department of Electronic Engineering, University of York, York, UK; b Seagate Technology,1 Disc Drive, Springtown Industrial Estate, Londonderry, Northern Ireland; c Institute for Materials Research, Tohoku University, Sendai, Japan; d Center for Spintronics Research Network, Tohoku University, Sendai, Japan; e Center for Science and Innovation in Spintronics, Core Research Cluster, Tohoku University, Sendai, Japan; f Research Center for Magnetic and Spintronic Materials, National Institute for Materials Science, Tsukuba, Japan; g Graduate School of Pure and Applied Sciences, University of Tsukuba, Tsukuba, Japan

**Keywords:** Heusler alloy, half-metallic ferromagnet, spin polarisation, magnetic moment, atomic disorder, curie temperature, minority bandgap, antiferromagnet, spin gapless semiconductor

## Abstract

Heusler alloys are theoretically predicted to become half-metals at room temperature (RT). The advantages of using these alloys are good lattice matching with major substrates, high Curie temperature above RT and intermetallic controllability for spin density of states at the Fermi energy level. The alloys are categorised into half- and full-Heusler alloys depending upon the crystalline structures, each being discussed both experimentally and theoretically. Fundamental properties of ferromagnetic Heusler alloys are described. Both structural and magnetic characterisations on an atomic scale are typically carried out in order to prove the half-metallicity at RT. Atomic ordering in the films is directly observed by X-ray diffraction and is also indirectly probed via the temperature dependence of electrical resistivity. Element specific magnetic moments and spin polarisation of the Heusler alloy films are directly measured using X-ray magnetic circular dichroism and Andreev reflection, respectively. By employing these ferromagnetic alloy films in a spintronic device, efficient spin injection into a non-magnetic material and large magnetoresistance are also discussed. Fundamental properties of antiferromagnetic Heusler alloys are then described. Both structural and magnetic characterisations on an atomic scale are shown. Atomic ordering in the Heusler alloy films is indirectly measured by the temperature dependence of electrical resistivity. Antiferromagnetic configurations are directly imaged by X-ray magnetic linear dichroism and polarised neutron reflection. The applications of the antiferromagnetic Heusler alloy films are also explained. The other non-magnetic Heusler alloys are listed. A brief summary is provided at the end of this review.

## Graphical abstract

**Figure:**
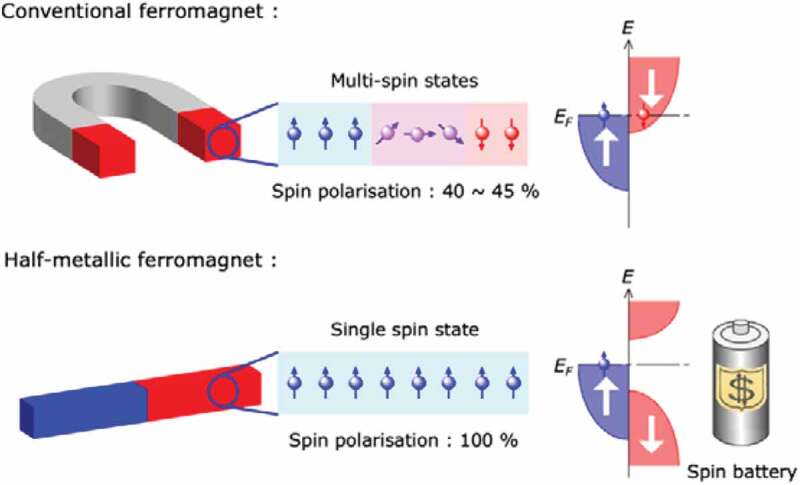

## 1. Introduction

Spintronics has been initiated by the discovery of giant magnetoresistance (GMR) by Fert et al. [1] and Grünberg et al. [2] independently. A GMR device consists of a sandwich structure of a ferromagnet (FM)/non-magnet (NM)/FM multilayer, where an external magnetic field can align the FM magnetisations in parallel to achieve a low-resistance state as compared with a high-resistance state with antiparallel magnetisations without a field application. The first-generation spintronic devices are based on magnetoresistive (MR) junctions, which have been used very widely [3,4], *e.g*., a read head in a hard disk drive (HDD) [5] and a cell in a magnetic random access memory (MRAM) [6]. The critical measure of efficient magnetic transport in these devices is an MR ratio, which is defined by^152^,^170^,^183^,^289^,^359^,^360^,^361^,^367^,^367^,^384^,^382^,^383^,^348^,^349^

MR ratio = Δ*R*/*R* = (*R*_AP_ – *R*_P_)/*R*_P_,(1)

where *R*_P_ and *R*_AP_ represent the resistance measured for parallel and antiparallel configurations of the ferromagnet magnetisations, respectively. The MR ratio determines the signal-to-noise ratio of these devices, which directly corresponds to the miniaturisation of them. To date, the maximum GMR ratio in the current-in-plane (CIP) geometry has been reported to be 65% at 300 K in a [Co (0.8)/Cu (0.83)]_60_ (thickness in nm) junction [7].

Similar MR changes have been demonstrated in a magnetic tunnel junction (MTJ) by replacing an NM layer with an oxide tunnelling barrier in a GMR junction [8]. Tunnelling magnetoresistance (TMR) at room temperature (RT) has then been achieved by Miyazaki and Tezuka [9] and by Moodera et al. [10] independently. Since then, the TMR ratio has been improved very rapidly to 81% in MTJ consisting of Co_0.4_Fe_0.4_B_0.2_ (3)/Al (0.6)-O*_x_*/Co_0.4_Fe_0.4_B_0.2_ (2.5) (thickness in nm) at RT [11]. By replacing amorphous AlO_x_ with epitaxial MgO, theoreticians have predicated over 1,000% TMR ratios due to coherent tunnelling via the ∆_1_ band matched at an Fe/MgO interface [12,13]. Here, the TMR ratio can be defined as [8]

TMR ratio = 2*P*_1_*P*_2_/(1-*P*_1_*P*_2_), (2)

where *P*_1(2)_ are effective spin polarisation of a ferromagnetic layer 1(2), respectively. For the coherent tunnelling, *P*_1(2)_ can be 100%, leading to the TMR ratio of infinity. Experimentally, giant TMR ratios have been reported by Parkin [14] and Yuasa [15] independently. Accordingly, a TMR ratio as large as 604% has been achieved in MTJ consisting of Co_0.2_Fe_0.6_B_0.2_ (6)/MgO (2.1)/Co_0.2_Fe_0.6_B_0.2_ (4) (thickness in nm) at RT [16]. Such a drastic increase in the TMR ratio has increased the areal density of HDD by almost four times over the last decade, for example [3].

**Figure 1.**
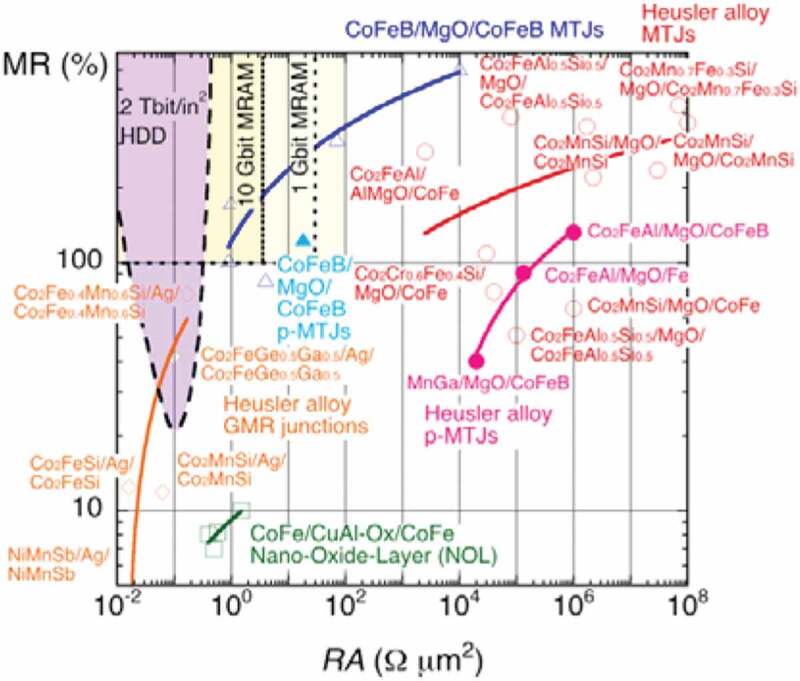
Relationship between a MR ratio and *RA* of MTJs with CoFeB/MgO/CoFeB (blue triangles), nano-oxide layers (NOL, green squares) and Heusler alloys (red circles) with in-plane (open symbols) and perpendicular magnetic anisotropy (closed symbols) together with that of GMR junctions with Heusler alloys (orange rhombus). The target requirements for 2 Tbit/in^2^ HDD read heads as well as 1 and 10 Gbit MRAM applications are shown as purple and yellow shaded regions, respectively. Reprinted with permission from Hirohata et al. [17]. Copyright 2018. MDPI AG

As shown in Figure 1 for 1 Gbit MRAM, the junction cell diameter (fabrication rule) should be <65 nm with a resistance area product (*RA*) <30 Ω·µm^2^ and an MR ratio >100% [18]. For 10 Gbit MRAM, the cell diameter should be reduced to be <20 nm with *RA* < 3.5 Ω·µm^2^ and an MR ratio >100%. Here, low *RA* is required to satisfy the impedance matching [19] and low power consumption (<100 fJ/bit). A standard MRAM architecture commercially employed is one MRAM cell with a transistor attached, where a large MR ratio (>150%) is essential to maintain a signal-to-noise ratio allowing for a read-out signal voltage to be detected by a small-current application. In order to achieve these requirements, the intensive investigation has been carried out on the CoFeB/MgO/CoFeB junctions. In-plane CoFeB/MgO/CoFeB MTJs have successfully satisfied the requirement for 10 Gbit MRAM by achieving *RA* = 0.9 Ω·µm^2^ and TMR = 102% at RT [20] as shown as open triangles with a blue fit in Figure 1. For further miniaturisation of the MRAM cells and the corresponding increase in the density, a perpendicularly magnetised MTJ (p-MTJ) has been investigated to achieve the requirement for 1 Gbit MRAM with *RA* = 18 Ω·µm^2^ and TMR = 124% at RT [21]. Further improvement has been made to satisfy 10 Gbit MRAM target [22], with satisfying a TMR ratio >100% and *RA* ~ 2 Ω·µm^2^. These MTJs under development are expected to replace the current-generation 256 Mbit MRAM with perpendicular magnetic anisotropy produced by Everspin [23]. Samsung shipped their new MRAM with a 28-nm fabrication rule for embedded-memory evaluation in March 2019 [24].

For a 2 Tbit/in^2^ HDD application, on the other hand, MTJ cannot be used as the requirement for *RA* is almost one order of magnitude smaller than that for 10 Gbit MRAM [25]. One attempt is a nano-oxide layer (NOL) developed by Toshiba, which restricts a current path perpendicular to a GMR stack by oxidising a part of the NM Cu or Al spacer layer [26]. A Co_0.5_Fe_0.5_ (2.5)/Al-NOL/Co_0.5_Fe_0.5_ (2.5) junction has demonstrated *RA* = 0.5 ~ 1.5 Ω·µm^2^ and MR = 7 ~ 10% at RT. These values are below the requirement for the 2 Tbit/in^2^ HDD, and hence further improvement in these junctions is crucial.

**Figure 2.**
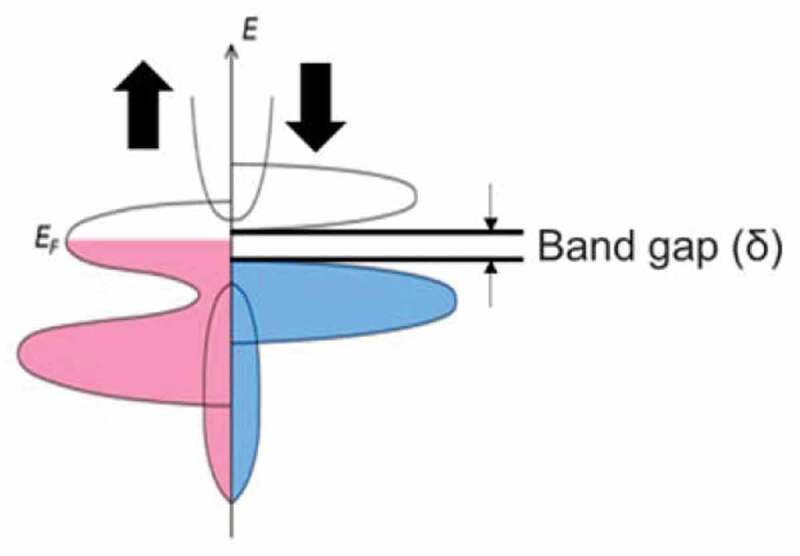
Schematic diagram of spin DOS for a HMF

For further improvement in the MR junctions to meet the requirements beyond 10 Gbit MRAM and 2 Tbit/in^2^ HDD, a half-metallic ferromagnet (HMF) needs to be developed to achieve 100% spin polarisation at the Fermi energy level (*E*_F_) at RT [27], leading to an infinite MR ratio using Eq. (1). The half-metallicity is induced by the formation of a bandgap *δ* only in one of the electron-spin bands in density of states (DOS) as schematically shown in Figure 2. There are four major types of HMFs theoretically proposed and experimentally demonstrated to date; (1) oxide compounds (*e.g*., rutile CrO_2_ [28] and spinel Fe_3_O_4_ [29]); (2) perovskites (*e.g*., (La,Sr)MnO_3_ [30]); and, (3) magnetic semiconductors, including Zinc-blende compounds (*e.g*., EuO and EuS [31], (Ga,Mn)As [32] and CrAs [33]) and (4) Heusler alloys (*e.g*., NiMnSb [34]). Among these HMFs, magnetic semiconductors have been reported to show 100% spin polarisation in a film form due to their Zeeman splitting in two spin bands. However, their Curie temperatures (*T*_C_) are still below RT [35]. Low-temperature Andreev reflection measurements have confirmed that both rutile CrO_2_ and perovskite La_0.7_Sr_0.3_MnO_3_ compounds possess almost 100% spin polarisation [36]; however, no experimental report has been proved the half-metallicity at RT to date. The Heusler alloys exhibit the half-metallicity at RT in a bulk form but not in an *ex situ* film form [37–39] but in an *in situ* film, (93 + 7/–11)% [40]. Therefore, the Heusler alloy films can be the most promising candidate for the RT half-metallicity due to their lattice constant matching with major substrates, high *T*_C_ and large *δ* at *E_F_* in general as detailed in the following sections.

### 2. Heusler alloys

**Figure 3.**
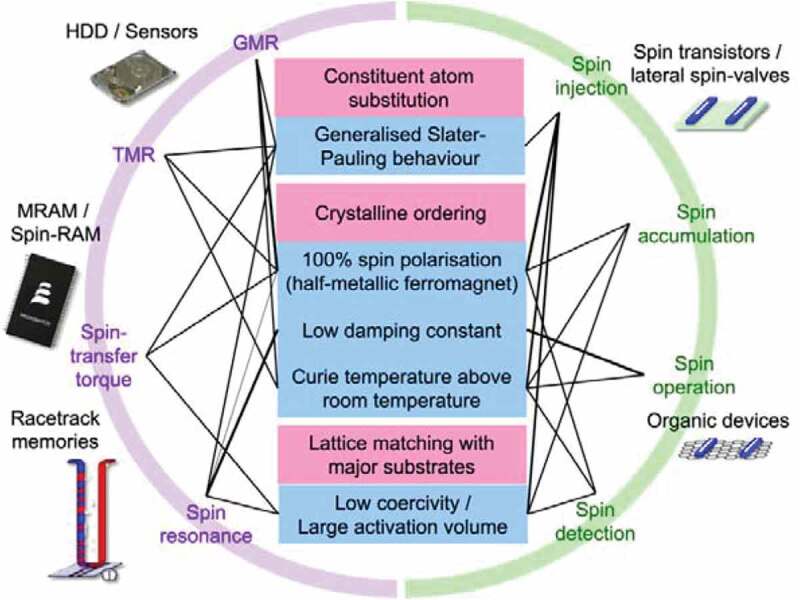
List of advantages and disadvantages of the Heusler-alloy usage in spintronic devices [41]. The width of the links represents the importance. Reprinted with permission from Hirohata et al. [17]. Copyright 2016

In order to employ the Heusler alloy films in spintronic devices, both advantages and disadvantages need to be considered as listed in Figure 3. Both structural and magnetic properties of the Heusler alloys can be controlled by the substitution of constituent elements of the alloy as detailed in the following sections. For example, the total spin magnetic moments, and the corresponding saturation magnetisation, can be precisely controlled by atomic substitution. Such controllability is useful for spin injection to minimise a stray field for the applications of an HDD read head, an MRAM cell and a magnetic racetrack memory, for instance. These properties also depend on the crystalline ordering of the Heusler alloys. For the half-metallicity, low damping constants and high Curie temperature, the perfect crystalline ordering needs to be achieved. Any departure from theoretical prediction on these properties can be attributed to disordering of the alloys. These magnetic properties are important for spin injection, accumulation, operation and detection in spintronic devices. Both structural and magnetic properties of the Heusler alloys are also depend on the lattice matching with substrates and seed layers. A low coercivity and large activation volume can be achieved by removing strain induced by lattice mismatch between them. These magnetic properties are again important for spin transport in devices.

### 2.1. Crystalline structures

**Figure 4.**
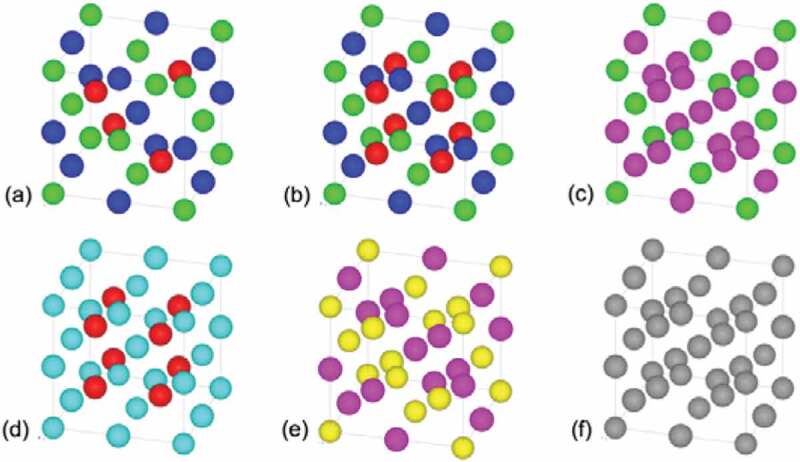
Crystalline structures of both (a) half- and (b) full- Heusler alloys; *C*1*_b_* and *L*2_1_ structures, respectively. Atomically disordered structures, (c) *D*0_3_, (d) *B*2, (e) *B*32*a* and (f) *A*2, are also shown. Data taken from Refs. [38,45,46]

**Figure 5.**
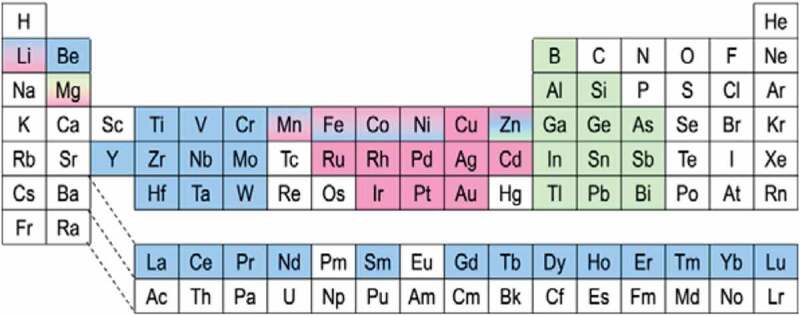
Periodic table showing typical X (blue colour), Y (pink colour) and Z (green colour) elements in Heusler compounds. Data are taken from Refs. [38,45,46]

Since the initial discovery of ferromagnetism in a ternary Cu_2_MnAl alloy, consisting of non-magnetic (NM) elements, by Heusler in 1903 [42], the Heusler alloys have been investigated intensively for various applications, including spintronic devices [37–39,41], magnetic refrigeration [43] and shape memory [44]. The Heusler alloys are categorised into two distinct groups by their crystalline structures; (1) half Heusler alloys with the form of XYZ in the *C*1*_b_* structure and (2) full Heusler alloys with the form of X_2_YZ in the *L*2_1_ structure as schematically drawn in Figure 4(a,b), respectively. Here, X and Y atoms are transition metals, while Z is either a semiconductor or an NM metal [see Figure 5] [34,45]. The unit cell of the *L*2_1_ structure consists of four face-centred cubic (fcc) sublattices, while that of the *C*1*_b_* structure is formed by removing one of the X sites. In the Heusler alloys, the half-metallicity is known to be fragile against atomic disorder. For the *L*2_1_ structure, when the Y and Z atoms replace their sites (Y-Z disorder) and eventually occupy their sites absolutely at random, the alloy transforms into the *B*2 structure [see Figure 4(c)]. Similarly, the X-Y disorder occurs to lead to the *D*0_3_ structure as shown in Figure 4(d). The mixture of X-Y and X-Z disorder forms the *B*32*a* structure [see Figure 4(e)]. In addition, X-Y and X-Z disorder finally forms the *A*2 structure as shown in Figure 4(f).

Recent studies propose the other classes of Heusler-alloy family, such as inverse [47,48] and equiatomic quaternary Heusler alloys [49,50]. The form of inverse Heusler alloys can be described as XYXZ for which one of X atom in the *L*2_1_ structure replaces with Y atom. Similarly, the form of equiatomic quaternary Heusler alloy can be described as XX’YZ for which one of X atom in the *L*2_1_ structure changes to the 4th element, X’. Interestingly, some of inverse Heusler alloys and equiatomic quaternary Heusler alloys also exhibit half-metallic electronic structure as well as electronic structure of spin gapless semiconductor (see Section 9.2).

**Figure 6.**
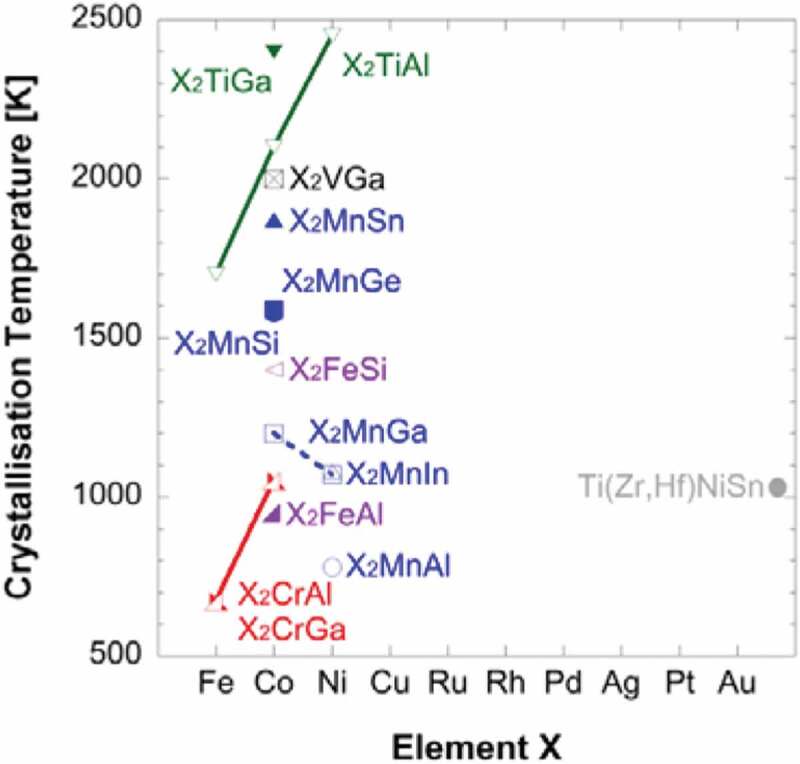
Crystalline ordering temperature of full-Heusler bulk alloys with respect to the element X. Experimental data are taken from Refs. [37,55,56]

Due to the complicated crystalline structures of the Heusler alloys as described above, they require very high temperature (typically >1000 K in the bulk form and >650 K in the thin-film form) for their crystalline ordering as shown in Figure 6 [51]. This fact hinders the use of Heusler alloy films to be used in spintronic devices. Recently, layer-by-layer crystallisation has been reported along the Heusler alloy (110) plane to reduce the crystallisation energy, resulting in the annealing temperature, by over 50% [52]. A similar crystallisation process has been demonstrated at a higher temperature to uniformly crystallise the Heusler alloy films [53]. Further reduction has been achieved using a W(110) seed layer, allowing over 80% *B*2 ordering by annealing at 355 K for 2 min [54]. Such layer-by-layer crystallisation can open a way for the implementation of a Heusler alloy film into spintronic devices.

**Figure 7.**
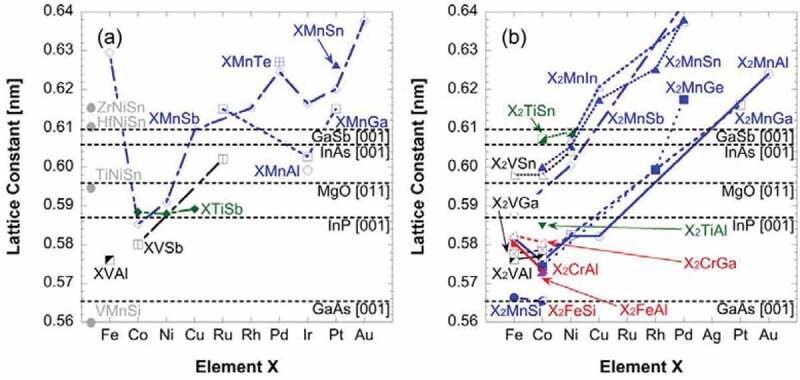
Lattice constant distribution of both (a) half- and (b) full-Heusler bulk alloys with respect to the element X. Experimental data are used from Refs. [41,45,57,58] and calculated data are taken from Refs. [59,60,61]. Lattice constants of major substrates are also shown as references

Most of crystallised Heusler alloys possess their lattice constants within the range of those of major substrates, clearly indicating the possibilities of epitaxial growth. Co-based full Heusler alloys especially hold excellent match with both GaAs(001) and MgO(001) substrates. The lattice constant can be precisely engineered to a required value by substituting a constituent element of the Heusler alloy X with a different atom as indicated as lines in Figure 7, and also by substituting the other elements Y or Z with the other atoms as categorised in Figure 7 with retaining the element X (*e.g*., Co_2_(Cr,Fe)Al). Such a crystallographical engineering approach is a powerful method to control the spin DOS in a unit cell to achieve robust half-metallicity at RT.

### 2.2. Magnetic properties

**Figure 8.**
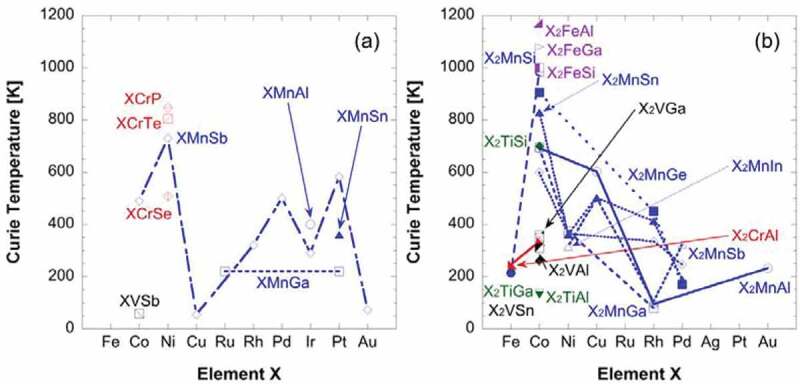
Curie temperature distribution of both (a) half- and (b) full-Heusler bulk alloys with respect to the element X. Experimental data are used from Refs. [41,45,57,58,61,62,66.] and calculated data are taken from Refs. [63–64]

The Curie temperature *T*_C_ of the Heusler alloys falls typically within the range between 200 and 1200 K as shown in Figure 8. *T*_C_ can also be further tuned to be well above RT by the atomic substitution as described in the previous section, achieving required spontaneous magnetisation at RT for the applications.

The robustness of the half-metallicity depends on the size and definition of the bandgap *δ* formed in one electron-spin band in the vicinity of Fermi energy level *E*_F_. *δ* is formed by the strong *d*-band hybridisation between the two transition metals of X and Y, according to *ab initio* calculations [67]. Typically, *δ* of 0.4 ~ 0.8 eV is expected to be formed at 0 K [38]. At a finite temperature, however, the bandgap becomes smaller and the edge definition of *δ* becomes broader. *δ* has been measured by detecting photon absorption of circularly polarised infrared light with energy corresponding to *δ* [64,68].

**Figure 9.**
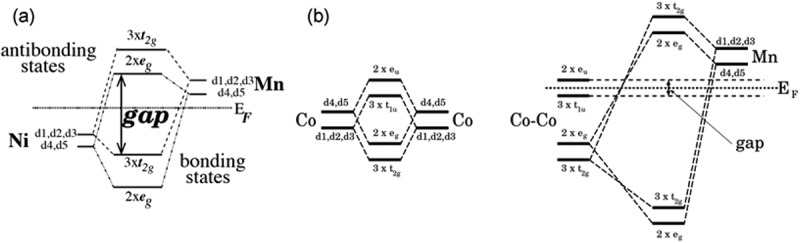
Schematic illustrations of the origin of the minority bandgap in (a) half- and (b) full-Heusler alloys (NiMnZ and Co_2_MnZ as examples, respectively). d1, d2, d3, d4 and d5 represent *d_xy_, d_yz_, d_zx_, d_z_*^2^ and *d_x_*^2^*_−y_*^2^ orbitals, respectively. Reprinted with permission from Galanakis et al. [69]. Copyright 2006. The Institute of Physics Publishing

The origin of *δ* in the Heusler alloys is attributed to the strong *d*-band hybridisation of the two elements X and Y. According to the calculations by Galanakis et al. [69], the local DOS in the vicinity of *E_F_* is dominated by the *d*-states, forming an energy gap between the higher degenerate of bonding hybridised states in the valence band and the lower degenerate of antibonding states in the conduction band. For the half-Heusler alloys [Figure 9(a)], the gap is formed between the hybridised states of the elements X and Y, *i.e*., between the three-fold degenerate (*t*_2*g*_) in the bonding states and the two-fold degenerate (*e_g_*) in the antibonding states. Therefore, most of the half-Heusler alloys possess an indirect bandgap between the valence band minimum at the Γ point and the conduction maximum at the X point. For the full Heusler alloys, on the other hand, the *d*-band hybridisation between the elements X plays a very important role, although these atoms occupy the second-nearest neighbour sites [see Figure 4(b)]. As shown in Figure 9(b), the X–X hybridisation initially forms both bonding and antibonding states for both *t*_2*g*_ and *e_g_*. The two bonding states among these four X–X orbitals then hybridise with the Y degenerates, developing both bonding and antibonding degenerates with a very large gap in between. The two X–X antibonding states, however, cannot couple with the Y degenerates, maintaining the small gap across *E*_F_, which defines the bandgap for the full-Heusler alloys. This can provide either a direct bandgap at the Γ point or an indirect bandgap between the Γ and X points.

**Figure 10.**
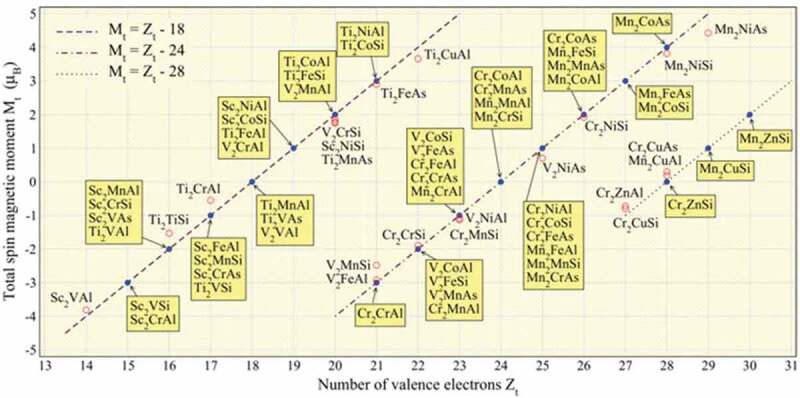
Total spin magnetic moments per unit cell (*M*_t_/f.u.) as a function of the total number of valence electrons in the unit cell for major Heusler alloys. The lines represent three different forms of the generalised Slater-Pauling curves. Reprinted with permission from Skaftouros et al. [74]. Copyright 2006. The American Physical Society

In the Heusler alloys, total spin magnetic moments per formula unit (f.u.) *M*_t_ have been reported to follow the generalised Slater-Pauling curve by Galanakis et al., which is represented as *M_t_* = *Z_t_* – 18 (half-Heusler) and *Z_t_* – 24 (full-Heusler), where *Z_t_* is the total number of valence band electrons as shown in Figure 10 [67,69]. This behaviour enables us to preferentially control the magnetic properties, the spin DOS at *E*_F_ in particular, continuously by substituting the Y atoms with the other transition metals as listed in Figure 5. Even though there are almost 3,000 possible combinations to form ternary Heusler alloys, there are about a few tens of alloys reported to become the HMFs according to theoretical calculations to date [see Tables 1 and 2]. For example, a Co_2_CrAl alloy has been theoretically calculated to become a HMF [70]; however, *T*_C_ has been reported to be around RT (334 K) for bulk [71]. In order to increase *T*_C_, the substitution of the Cr atoms with the Fe atoms has been successfully reported experimentally [72,73], proving the spin engineering by crystallographical manipulation.

**Table 1.** List of major magnetic properties of half-metallic half-Heusler alloys. Italic and roman letters correspond to calculated and experimental values, respectively. * indicates calculated total magnetic moments per formula unit, while the others are calculated spin magnetic moments per formula unit

Half Heusler alloys	Magnetic moment [μ_B_/f.u.]		*δ* [eV]	*T*_C_ [K]	*P* [%]	Refs.
	*Calculation*	Experiment				
NiMnSb	*4.00* [34]	3.85 (bulk) [45,88]	*0.5* [69]	*900* *~1112* [100]	*99.3* [82]	[78,79,82,85–87,90–95]
	*3.9910* [75]	3.6 (poly) [89]	0.5 [83]	730 (bulk) [45]	58 ± 2 [36]	
	*3.991* [69]*	3.9 ± 0.2 [84]	0.55 [61]		~ 44 [89]	
	*3.96* [70,87]	4.01 ± 0.02 [87,90]			~ 100 [76,77]	
PtMnSb	*4.00* [34]	4.14 (bulk) [45]		582 (bulk) [45]		[92,96]
	*3.997* [69]*	3.97 (bulk) [88]				[99]
FeMnSb	*1.930* [97]* [69]*		*0.436* [60]		*99.3* [75]	
	*2.000* [98]					
CoMnSb	*2.949* [67]* [69]*	4.0 (bulk) [45]	*~ 1* [69]	*671* *~815* [100]	*99.0* [75]	[88]
	*3.000* [99]	3.00 [61]		490 (bulk) [45]		
	*3.03* [61]					
CoTiSb			*0.95* [101]			
			*0.82* [69]			
			*1.03* [102]			
NiTiSb	*0.0* [101]		*0.14* [69]	330 (bulk) [70]		
FeVSb			*0.36* [69]			
			*0.38* [63]			
CoZrSb			*0.83* [69]

**Table 2.** List of major magnetic properties of full-Heusler alloys. Italic and roman letters correspond to calculated and experimental values, respectively. * indicates calculated total magnetic moments per formula unit, while the others are calculated spin magnetic moments per formula unit. Spin-polarisation values with † and †† are estimated at RT and 2 K, respectively, by using Jullière’s formula [8]. Several non-half-metallic full Heusler alloys are also shown in brackets as references

Full Heusler alloys	Magnetic moment [μ_B_/f.u.]		*δ* [eV]	*T*_C_ [K]	*P* [%]	Refs.
	*Calculation*	Experiment				
Co_2_MnAl	*4.09* [106]	4.01 (bulk) [45]	*0.306* [109]	693 (bulk) [45]	42 [110]†	[112]
	*4.10* [107]				58 [111]†	[113]
	*3.970* [67]				60 [62]	[114]
	*4.020* [108]					
	*4.036* [69,97] *					
Co_2_MnSi	*5.00* [106]	5.07 (bulk) [45]	*0.419* [109]	*740 ~ 857* [100]	54 ± 3 [117]	[114]
	*4.96* [115]	5.10 ± 0.04 (bulk) [116]	*0.81* [121]	985 (bulk) [45]	35 [122]†	
	*4.940* [67]	4.95 ± 0.25 [117]		690 [57]	89 [122]††	
	*5.0* [116]	4.7 [118]		900 [62]	56 [62]	
	*5.008* [69,97] *	5.0 [119]				
		5.1 [120]				
Co_2_MnGa	*4.14* [106]	4.05 (bulk) [45]		694 (bulk) [45]	~ 50 [123]	[124]
	*4.21* [107]			700 [62]	60 [62]	
	*3.72* [115]					
	*4.058* [67]					
Co_2_MnGe	*5.00* [106]	5.11 (bulk) [45]	*0.210* [109]	905 (bulk) [45]	58 [62]	[105]
	*4.84* [115]	4.93 (bulk) [125]	*~ 0.5* [106]	900 [62]		[126]
	*4.941* [67]	5.1 [104]				
	*5.0* [120]					
	*5.012* [69,97] *					
Co_2_MnSn	*5.03* [106]	5.08 (bulk) [45]	*0.174* [109]	829 (bulk) [45]	60 [62]	[114]
	*4.78* [115]			800 [62]		
	*4.984* [67]					
	*5.0* [116,120]					
	*5.043* [108]					
	*5.089* [69,97] *					
Co_2_CrAl	*2.99* [106]	1.55 (bulk) [45]	*0.18* [87]	334 (bulk) [45]	*~ 100* [87]	[131]
	*2.955* [67]	1.5 ~ 3 [129]	*0.75* [127]	330 [130]	*~97* [131]	[132]
	*3.007* [108]	0.53 [130]	*0.18* [128]	600 [62]	*~98* [128]	[133]
	*3.0* [87]				16 [132]†	[134]
	*2.999* [69] *				62 [62]	[135]
	*2.96 ~ 3.01* [127]					
	*2.970* [128]					
Co_2_Cr_0.6_Fe_0.4_Al	*3.7* [131]	3.4 (bulk) [136]	*~ 0.4* [127]	750 (bulk) [136]	*~ 90* [141]	
		3.65 (bulk) [137]		665 ± 2 (bulk) [137]	*~ 95* [128]	
		2.04 [73]			18 [73]†	
		3.5 [129]			25 [137]†	
		3.1 [132]			29 [132]†	
		3.2 (RT) (bulk) [138] 3.49 (bulk) [139]				
		3.3 [140]				
		2.26 (RT) [38]				
(Co_2_FeAl)	*4.996* [106]	4.9 [129]	*0.1* [127]	1170 (bulk) [61]	*~ 30* [141]	[131]
	*4.98* [107]	4.8 [130]			46 [132]†	[132]
	*4.98* [67]	5.29 [139]			59 [62]	[135]
	*4.996* [69]*					
	*4.811* [128]					
Co_2_CrGa	*3.01* [106]	3.01 (bulk) [141]		495 (bulk) [141]	*95* [141]	
					61 [62]	
Co_2_FeSi	*5.28* [142]	*0.35* [142]		*1109* [143]	16 [145]	[150]
	*5.48* [143]	*0.10* [143]		1100 [144]	57 ± 1 [147]	[151]
	*6.0* [144]	*~ 0,15* [144]		1100 [62]	42 [148]	[153]
	5.97 ± 0.05 [144]				74 [148]	[154]
	6.00 [145]				60 [62]	[155]
	5.5 [146]					
	5.73 [147]					
	4.36 ± 0.55 [148]					
	5.56 [148]					
	4.8 [149]					
Co_2_FeGa				1100 [62]	58 [62]	
Co_2_FeGe				1000 [62]	58 [62]	
Co_2_NiGa		3.21 (bulk) [45]		670 [57]		
Co_2_TiAl	*1.00* [156]		*0.438* [156]	135 (bulk) [157]		
				126 [66]		
Co_2_TiSi	*2.00* [156]		*0.800* [156] *0.62* [143] *0.64* [157]			
Co_2_TiGa	*1.00* [156]		*0.157* [156]			
Co_2_TiGe	*2.00* [156]		*0.602* [156]			
Co_2_TiSn	*1.85* [115]	1.93 (bulk) [45]	*0.478* [156]	*359* [115]	57 [62]	
	*1.784* [67]	1.92 (bulk) [158]	0.0123 [159]	359 (bulk) [45]		
	*2.00* [156]			~ 350 (bulk) [159]		
	*1.68* [158]			364 [62]		
Co_2_VAl					48 [62]	
Co_2_VSi	*3* [[143]		*0.68* [143]	*566* [[143]		
Co_2_ZrAl				194 [66]		
Co_2_ZrSn	*1.64* [158]	1.64 (bulk) [158]				
Co_2_NbSn	*1.08* [158]	0.94 (bulk) [158]				
(Co_2_TiSb)	*1.73* [156]		*0.567* [156]			
Co_2_FeGa	*5.15* [115]			> 1100 [115]		
(Ni_2_MnAl)				~ 350 [160]		
(Ni_2_MnGa)				~ 320 [161]		[164]
				~ 350 [162]		
				~ 340 [163]		
(Ni_2_MnGe)				~ 320 [163,165,166]		
(Ni_2_MnIn)				~ 170 (*B*2) [166]		
				318 (*L*2_1_ and *B*2) [167]	34 [167]††	[168]
				320 ~ 323 [66]		
(Ni_2_MnSb)				334 [66]		
(Ni_2_MnSn)				344 [66]		
(Cr_2_MnP)	*0.102*			240		[169]
(Cr_2_MnAs)	*0.096*			250		[169]
Cr_2_MnSb	*0.036*			342		[169]
(Cr_2_MnBi)	*–0.011*			320		[169]
(Fe_2_VAl)					56 [62]	
(Pd_2_MnSb)				247 [66]		
(Pd_2_MnSn)				189 [66]		

### 3. Ferromagnetic Heusler alloys

#### 3.1. Half-Heusler alloy films

Since the pioneering theoretical prediction on the half-metallicity of a NiMnSb half-Heusler alloy [34], this alloy has been intensively investigated to confirm its half-metallicity experimentally. As listed in Table 1, the magnetic moment per formula unit and the bandgap *δ* are calculated as 3.99 μ_B_/f.u. and 0.5 eV [69], respectively. The corresponding spin polarisation is calculated to be 99.3% [75]. For a bulk single crystal, the NiMnSb alloy has indeed been measured to show almost 100% spin polarisation at *E*_F_ by means of spin-polarised positron-annihilation [76,77]. Both ultrahigh vacuum (UHV) co-sputtering [78] and molecular beam epitaxy (MBE) [79] techniques are employed to grow epitaxial films, which are characterised mostly by X-ray diffraction (XRD) and magnetocrystalline anisotropy measurements. However, the spin polarisation is found to be only 28% at 0.4 K estimated from TMR [79]. For the NiMnSb film grown in a similar manner, the surface spin polarisation *P* is measured to be only ~58% [36]. This large departure from the bulk property can be explained by the presence of the atomic disorder at the empty sites [80]. *δ* for the minority spins at *E*_F_ is reported to vanish with an atomic disorder of more than 7%. In addition, the surface state is very fragile due to the reduced symmetry and the surface reconstruction [81,82]. On the contrary, epitaxial NiMnSb (100) films grown on Mo(100) buffers on MgO(111) substrates have shown (67 ± 9) to 100% spin polarisation at the MnSb terminated surface, which is much higher than that of 50% for polycrystalline samples measured by angle-resolved X-ray photoemission [83]. A study on epitaxial NiMnSb(001) growth on MgO(001) has also been performed with using a V(001) buffer layer by Turban et al. [84]. They find the Stransky-Krastanov growth mode under the optimised growth temperature of 620 K. The corresponding magnetic moment is estimated to be (3.9 ± 0.2) μ_B_/f.u., which agrees almost perfectly with the calculations [34,69].

Epitaxial NiMnSb(001) growth on GaAs(001) has been studied systematically by van Roy et al. [85]. The films are grown at RT (300 K) to avoid interfacial Mn_2_As mixing between NiMnSb and GaAs, which may introduce a magnetically dead layer. The lattice constant is slightly enhanced to 0.5904 ~ 0.5909 nm as compared with the bulk value (0.5903 nm) due to the small lattice mismatch with GaAs. Stoichiometric epitaxial NiMnSb films are also grown on GaAs(111)B [86]. These results clearly indicate that the presence of the vacancy sites in the half-Heusler alloy [see Figure 4(a)] does not contradict high chemical ordering under precisely controlled deposition. They also find that these films contain very small point defect concentrations: 1.1% Mn planer defects appear in a 1 nm thick region in the vicinity of the NiMnSb/GaAs:B interface, which agrees very well with calculations that the half-metallicity can be recovered within less than 6 atomic planes (≤0. 7 nm) [91]. For the NiMnSb interfaces, the lattice mismatch is calculated to induce strain: 2% lattice expansion reduces *δ* by 0.25 eV, while 2% lattice compression increases *δ* by 0.5 eV [88], and the −2 to +3% lattice expansion maintains the half-metallicity [87].

Besides the possible disappearance of the half-metallicity due to the atomic disorder and the interfacial mixing, which can be overcome by the optimised growth as mentioned above, the surface spin polarisation is calculated with respect to the interfaces, the crystalline orientations and the terminated lattice planes. For the NiMnSb(001) surfaces, the Ni-terminated surface compresses the distance between the topmost Ni atoms and the subsurface MnSb layers by 10% (*P* = 42%), while the MnSb-terminated surface reduces the distance between the topmost Mn and the subsurface Ni layers by 3.5% and expands that between the topmost Sb and the subsurface Ni layers by 7.3% (*P* = 84%) [94]. The NiMnSb(111) surfaces, on the other hand, show much larger surface deformation: The Ni-terminated surface shows 23% and 18% reduction in the Ni-Sb and Ni-Mn distances, respectively, reducing the corresponding *P* significantly. For the case of a NiMnSb/InP interface, the Mn surface is calculated to increase the spin moment up to 4.0 μ_B_, inducing the corresponding spin polarisation of about 74%, while the Ni/P interface reduces the spin polarisation down to 39% for the first two interfacial layers [95].

Similar argument can be applied for the other half-Heusler alloy films. PtMnSb films are deposited on Al_2_O_3_(0001) [96,99] and MgO(001) [103] by sputtering to form spin-valve structures, showing <1% giant magnetoresistance (GMR) at RT. This small GMR ratio may also be due to the empty site disorder at the interfaces. Calculations suggest the decrease in the surface spin polarisation depends on the terminated layers; *P* ~ 46% and 22% for the MnSb and Pt termination, respectively, [92]. The other half-Heusler alloy of CoMnSb shows a similar decrease in the surface spin polarisation and the bandgap by the strain: +2% and – 2% lattice deformation shifts *δ* by – 0.8 eV and + 0.9 eV, respectively [94].

### 3.2. Full-Heusler alloy films

#### 3.2.1. Co-based full-Heusler alloys

##### (i) Co_2_MnZ

A pioneering work on the growth of full-Heusler-alloy films has been carried out for a Co_2_MnGe/GaAs(001) hybrid structure by Ambrose et al. [104]. They obtain an epitaxial Co_2_MnGe film with a slightly enhanced lattice constant as compared with bulk. The magnetic moment is estimated to be 5.1 μ_B_/f.u., which almost perfectly agrees with the bulk value [see Table 2]. For this alloy, calculations suggest that the strong reduction in the magnetic moment may occur near the Co_2_MnGe/GaAs interface due to the Co-As and Co-Ga bonding [105].

Consequently, systematic search has been widely performed over Co_2_Mn-based full Heusler alloys to realise the half-metallicity at RT; Co_2_MnAl [110–114], Co_2_MnSi [115–117,118–120,122], Co_2_MnGa [123,124] and Co_2_MnSn [114]. For Co_2_MnAl, an epitaxial film has been grown on a Cr buffer layer by UHV sputtering with the crystalline relationship of Co2MnAl(001)[110]||Cr(001)[110]||MgO(001)[100] but with the *B*2 structure [112]. For Co_2_MnSi, the *L*2_1_ structure has been deposited using both UHV dc magnetron sputtering [117,118,120,122] and MBE techniques [119]. Some of these films are used as electrodes in MTJ and recently show large TMR ratios at low temperature, which is discussed in Sec. 4.3.

Calculations imply that the strain induced in the unit cell can control the half-metallicity in the Co_2_MnZ alloys as similarly discussed for the half-Heusler alloys in Sec. 3.1. For Co_2_MnSi for example, the lattice compression of 4% increases *δ* by 23%, and a similar behaviour is expected for the other alloys [121]. Calculations also show that ± 2% change in the lattice constant preserves the half-metallicity in the Co_2_MnZ alloys [69].

##### (ii) Co_2_(Cr,Fe)Al

Block et al. have presented large negative magnetoresistance (MR) at RT in a quaternary full Heusler Co_2_Cr_0.6_Fe_0.4_Al alloy [136], which firmly proves the controllability of the spin DOS of the Heusler alloys by substituting their constituent elements. They report – 30% MR at RT for a pressed powder compact, which acts as a series of MTJs. As a result, a great amount of attempts has been made to utilise this alloy system to achieve a large MR ratio at RT due to the half-metallicity [73,129,132,137]. However, an epitaxial film is deposited on a MgO(001) substrate with the crystalline relationship of Co2Cr0.6Fe0.4Al(001)[100]||MgO(001)[110], showing only 2% GMR at RT (4% at 15 K) [129].

The influence of the atomic disorder on the half-metallicity for the Co_2_Cr_1-*x*_Fe*_x_*Al full Heusler alloys has also been systematically studied by Shirai et al. using first principles calculations [131,132,141]. In the Co_2_CrAl alloys, the atomic disorder between Cr and Al, which eventually deforms the crystalline structure from *L*2_1_ into *B*2 at a disorder level of 0.5, maintains the very high spin-polarisation *P* of 97% for *L*2_1_ and 93% for *B*2 [131]. The Co-Cr type disorder, however, destroys the half-metallicity rapidly: *P* falls to zero at a disorder level of 0.4 and the magnetic moment *M*_t_ reduces down to 2.0 μ_B_/f.u. at the full disorder. For the Fe substitution *x* with Cr, *P* is calculated to stay above 90% up to *x* = 0.35. Similarly, in the Co_2_Cr_1-*x*_Fe*_x_*Al alloys, the CrFe-Al type disorder preserves both *P* and *M*_t_ to be above 80% and 3.7 μ_B_/f.u., respectively, up to the disorder level of 0.5, while the Co-CrFe disorder eliminates *P* at the disorder level of 0.3 [132]. These findings may explain the decrease in the estimated *M*_t_ in the earlier study [73].

Strain also affects the half-metallicity in the Co_2_CrAl alloy according to calculations [72]. *P* stays ~ 100% in the lattice strain range between – 1 to + 3%, and is even higher than 90% under +10% strain. *δ* is also robust against the strain and can be maximised under +3% strain. *P* also remains ~100% against the tetragonal distortion in the range of ± 2%, which is a great advantage for the epitaxial growth study on major substrates.

Experimentally, stoichiometric epitaxial Co_2_Cr_1-*x*_Fe*_x_*Al films are directly grown on a GaAs(001) substrates using three-source co-evaporation with an ultrahigh vacuum (UHV) MBE technique, resulting in polycrystalline Co_2_CrAl/GaAs and epitaxial *L*2_1_ Co_2_FeAl(001)/GaAs(001) hybrid structures [130]. The Co_2_FeAl film grown at 673 K forms an almost perfect *L*2_1_ structure for the thickness above 7.5 nm, of which crystalline relationship is Co_2_FeAl(001)<110>||GaAs(001)<110> with showing strong uniaxial anisotropy along the [1–10] direction with a magnetic moment of 4.8 μ_B_/f.u. Even though the TMR ratio for this film is only 9% at RT, the growth condition for this Heusler alloy system has been successfully optimised. For intermediate states, *e.g., x* = 0.4, the *A*2 structure appears below the thickness of 1.2 nm [2 monolayers (MLs)], followed by the formation of the *B*2 and then the *L*2_1_ structures above 2.0 nm (3.5 MLs).

### 3.2.2. Ni-based full-Heusler alloys

Even though Ni_2_MnZ alloys are not predicted to become HMFs by calculations, detailed studies on epitaxial growth on GaAs and InAs have been reported by Palmstrøm [57]. By employing a Sc_0.3_Er_0.7_As buffer layer on GaAs(001), both Ni_2_MnAl [160] and Ni_2_MnGa [161–164] films are epitaxially grown. Although Ni_2_MnGa films are also epitaxially grown directly on GaAs(001) with the crystalline relationship of Ni2MnGa(001)[100][010]||GaAs(001)[100][010], no strong in-plane magnetocrystalline anisotropy is observed in their magnetisation curves [164]. Ni_2_MnGe(001)/GaAs(001) [163,165] and Ni_2_MnIn(001)/InAs(001) [166] hybrid structures are additionally fabricated for evaluation. Their interfaces are reported to be very sensitive to the growth temperature: Interfacial mixture occurs at the growth temperature of 373 K, while a large number of planer defects are formed at 433 K for Ni_2_MnGe/GaAs [163]. All these films are slightly tetragonally elongated along the plane normal as compared with the bulk values due to the minor lattice mismatch with the semiconductor substrates, and eventually the Ni_2_MnIn film on InAs transforms into the *B*2 structure. First principles calculations demonstrate that a broad energy minimum of tetragonal Ni_2_MnGa can explain stable pseudomorphic growth of Ni_2_MnGa on GaAs despite a nominal 3% lattice mismatch [161]. An antiferromagnetic (AF) phase of the Ni-based full-Heusler alloys is discussed in Sec. 5.2.

### 4. Major characterisations techniques of ferromagnetic Heusler alloys

#### 4.1. X-ray diffraction

For the structural analysis on the Heusler alloys, XRD is the most commonly used technique. XRD analysis predominantly focuses on both superlattice peaks, (111) and (200), and the principal peak (220). The appearance of all three peaks corresponds to the formation of the *C*1*_b_* or *L*2_1_ structures in the Heusler-alloy films, while the disappearance of the (111) peak represents the formation of the *B*2 structure and the disappearance of the two superlattice peaks indicates the formation of the *A*2 structure for the full-Heusler alloys. For the *L*2_1_ structure of the X_2_YZ full-Heusler alloys, the structure amplitudes of the XRD (111), (200) and (220) peaks are given by *F* (111) = 4 | *f*_Y_ – *f*_Z_ |, *F* (200) = 4 | 2 *f*_X_ – (*f*_Y_ + *f*_Z_) | and *F* (220) = 4 | 2 *f*_X_ + (*f*_Y_ + *f*_Z_) |, respectively, where *f*_M_ (M = X, Y and Z) are the average scattering factors for the M atoms [162]. The principal reflection (220) satisfies the relationship (*h* + *k* + *l*)/2 = 2 *n* (*h, k* and *l*: Miller indices, and *n*: an integer number), and is not affected by the atomic disorder. When the disorder occurs absolutely randomly among the M atoms, the magnitude of the first two superlattice peaks are reduced by the factor *S*^2^, where *S* is the degree of long range ordering described with the number of the X atoms on the *L*2_1_-ordered X sites *n*_X_ as *S* = {*n*_X_ – *n*_X_ (*A*2)}/{*n*_X_ (*L*2_1_) – *n*_X_ (*A*2)} (*S* = 1 for the *L*2_1_ structure). When the Y-Z disorder occurs, the second superlattice peak (200) with (*h* + *k* + *l*)/2 = 2 *n* + 1 is not affected, while the first peak with *h, k* and *l* are all odd is reduced by a factor of (1–2*a*) *S*^2^, where *a* is a disorder parameter defined as the fraction of the Y atoms occupying the Z sites (*a* = 0.5 for the *B*2 structure). By applying the structure amplitudes *F*(*h k l*), the XRD peak intensity *I*(*h k l*) can be calculated as follows: *I* (*h k l*) = | *F* (*h k l*) | ^2^ *p* {(1 + cos ^2^ 2*θ*)/sin ^2^ *θ* cos *θ*} (*p*: multiplicity factor) [171]. For the polycrystalline Co_2_CrAl alloy for instance, the peak intensity ratio normalised by the principal (220) reflection is calculated to be *I* (111): *I* (200): *I* (220) = 5: 6: 100, while it is 7: 5: 100 for the polycrystalline Co_2_FeAl alloy [130]. Comparison of these calculated values with experimental observations provides a measure of the atomic ordering in the Heusler alloy samples, however, cannot be applied directly to the epitaxial films.

Takamura et al. demonstrated a quantitative analysis method of atomic disorders in Co_2_FeSi Heusler alloy using XRD. It is capable to evaluate all the atomic disorders for the exchanges X, Y and Z atoms in full-Heusler X_2_YZ. Such technique relies on the use of different atomic scattering factors (*f*_Co_ and *f*_Fe_) of Co and Fe for Co *K*α source. Hence, superlattice diffraction intensities for Co *K*α can reflect the *D*0_3_ disorder [172]. A physical model proposed by Niculescu et al. was used so that the disorder parameters can be expressed in terms of α, β and γ. These parameters represent the quantitative disorder of *B*2 (atom mixing between Fe and Si), *A*2 (atom mixing between Co and Si) and *D*0_3_ (atom mixing between Co and Fe) disorder, respectively [173].

### 4.2. Cross-sectional transmission electron microscopy

Gabor et al. studied the correlation between the structural, electronic and magnetic properties of Co_2_FeAl_0.5_Si_0.5_ epitaxial films [174]. Cross-sectional transmission electron microscopy (TEM) was employed to determine the crystalline structures of the films. Bright field images show that the sample has homogeneous roughness and thickness. Single-crystalline *B*2 structure is confirmed by the selected area diffraction pattern. In 2006, Sakuraba et al. illustrated the correction between the crystallinity of Heusler alloy films and the TMR ratios [111]. Epitaxial and polycrystalline Co_2_MnAl films were imaged using bright field cross-sectional TEM. It is important to mention that the interfacial roughness between the Co_2_MnAl and AlO is greatly reduced for the epitaxial Co_2_MnAl film. Electron diffraction patterns show the disordered *B*2. Temperature-dependant MR measurements were also performed from 10 to 300 K. In general, epitaxial Co_2_MnAl shows about 15% higher MR ratio than the polycrystalline Co_2_MnAl, confirming the importance of the crystalline ordering on the spin polarisation and the corresponding MR ratios. Yamada et al. demonstrated the interface crystallinity of Co_2_FeSi as a function of growth temperature [175]. Two-dimensional epitaxial structure is observed at the growth temperature of 333, 403 and 473 K. However, at 403 and 473 K single-crystal phases are observed using cross-sectional TEM. Nanobeam diffraction confirms the phases may be CsCl- and/or CaF_2_-type silicides such as nonmagnetic CoSi, FeSi and/or CoSi_2_ [176].

These studies demonstrated structural characterisation on Heusler thin film samples using a cross-sectional TEM method. It also reveals the chemical distributions and crystalline ordering of the Heusler alloy films. However, some Heusler alloys form multiple phases instead of a single ordering structure, and their ratio is difficult to estimate.

### 4.3. Electrical resistivity

As the other macroscopic measure to assess the half-metallicity, electrical resistivity is commonly measured as a function of temperature, *ρ* (*T*). In general, the temperature dependence of the resistivity can be written as *ρ* (*T*) = *ρ* (4 K) + *c T ^m^*, where *m* is an exponent factor. In a conventional FM metal, since one-magnon scattering (or electron-electron scattering) dominates the resistivity at low temperature, *m* becomes 2 theoretically [177]. For HMF, due to the 100% spin polarisation, the one-magnon scattering is suppressed by the factor of exp (– *δ*/*k*_B_ *T*) (*δ* is energy bandgap for the minority spins at *E*_F_ and *k*_B_ is Boltzmann constant), leading to typically *m* = 1.5 at low temperature. At a finite temperature, spin fluctuation activates the minority band and unconventional one-magnon scattering starts to happen, which is described as *m* = 3.

Experimentally, *ρ* (*T*) of an epitaxial NiMnSb film follows a *T* ^1.55^ law below 100 K as listed in Table 3, which clearly indicates the absence of spin-flip electron diffusion due to the half-metallicity [84]. For the full-Heusler alloys, on the other hand, *ρ* (*T*) is observed to be almost constant at low temperature, while *m* is measured to be *m* = 1.5 and (1.2 ± 0.1) at a finite temperature below RT in single-crystal Co_2_MnGe [104] and polycrystalline Co_2_MnGa films [124], respectively. Co_2_CrAl bulk similarly shows *m* = 3.15 at low temperature but 1.33 above 35 K [134]. Epitaxial Co_2_FeAl films show *m* = 2.6 below 50 K but 1.3 above 100 K [129], and *m* = 4.2 below 30 K but 1.5 above 115 K [130]. For Co_2_MnSi films [117], a relationship of *ρ* (*T*) = *T* ^2^ + *T* ^4.5^ is reported, which may consist of an electron-electron scattering term *T* ^2^ and a two-magnon scattering term *T* ^4.5^. Such a departure from the ideal law observed in the full-Heusler alloys, especially at low temperature, is mainly attributed to the presence of grain boundaries in the films, for the case of the polycrystalline films in particular [116].

**Table 3.** List of exponent factors in the temperature dependence of the electrical resistivity *ρ* (*T*) = *ρ* (4 K) + *c T ^m^* for both half- and full-Heusler alloys. The residual resistivities (RRR) *ρ* (*T*)/*ρ* (4 K) are also shown

	*m* at low temperature	*m* at high temperature	RRR	Refs.
Theory	1.5 (HMF) 2.0 (conventional FM)	3.0 (non-rigid band) 4.5 (rigid band)		[177]
NiMnSb	1.55 (< 100 K)			epi-*C*1*_b_*-MBE film/V seed/MgO(001) sub. [84]
Co_2_MnGe	0 (< 50 K)	~ 1.5 (> 50 K)	~ 1.28	epi-*B*2-MBE film/GaAs(001) sub. [101]
Co_2_MnGa	0 (< 100 K)	(1.2 ± 0.1) (> 200 K)	1.15 ~ 1.7	poly-(*B*2)-MBE film/GaAs(001) sub. [124]
	2.1 (< 60 K)	1.31	2.47	Bulk [178]
Co_2_Cr_0.6_Fe_0.4_Al	2.6 (< 50 K)	1.3 (> 100 K)		epi-(*B*2)-sputtered film/MgO(001) sub. [129]
Co_2_CrAl	3.15 (< 35 K)	1.33 (> 35 K)	1.1	*L*2_1_-bulk [134]
			< 1	poly-*B*2-MBE film/GaAs(001) sub, [130]
Co_2_FeAl	2.6 (< 50 K)	1.3 (> 100 K)		epi-(*B*2)-sputtered film/MgO(001) sub. [129]
	4.2 (< 30 K)	1.5 (> 115 K)	1.3	epi-*L*2_1_-MBE film/GaAs(001) sub. [130]
Co_2_MnSi			6.5	single-crystal bulk [116]
		2.22 (> 75 K)	1.367	*L*2_1_-bulk [179]
	2 (< 100 K)	4.5 (> 100 K)	1.41	poly-*L*2_1_-sputtered film/*a*-Al_2_O_3_(0001) sub. [117]
Co_2_FeSi	3.5 (< 70 K)	1.65 (> 70 K)	1.5	epi-*L*2_1_-sputtered film/MgO(001 sub. and Al_2_O_3_(11–20) sub. [149]
Co_2_TiAl	2 (< 100 K)		4.2	*L*2_1_-bulk [157]

The residual resistivity ratio (RRR), *ρ* (300 K)/*ρ* (4 K), can also be used to characterise the bulk properties of the half-metallic films. For Co_2_MnGa [124] and Co_2_CrAl [130], the normalised resistivity *ρ* (*T*)/*ρ* (4 K) has been reported to decrease monotonically with increasing *T*, providing RRR to be less than 1, which is common for a highly resistive material, such as an intrinsic semiconductor. For most of the Heusler films, RRR is obtained to be approximately 1.3, *e.g*., 1.28 for a single-crystal Co_2_MnGe film [104] and 1.3 for epitaxial *L*2_1_ Co_2_FeAl film [130], which is much smaller than that observed for a Co_2_MnSi bulk single crystal (6.5) [116] and for Co_2_TiAl bulk (4.2) [157]. Since a very large RRR is reported for the bulk single crystal due to the improvement of the crystallinity of the alloy at low temperature, small RRRs for the Heusler films may indicate the stable crystallinity against temperature change. By comparing RRR with the *ρ* (*T*), an epitaxial Heusler alloy film without grain boundaries is expected to show the ideal *ρ* (*T*) behaviour.

### 4.4. X-ray magnetic circular dichroism

As a direct method to estimate the element specific magnetic moments per atom, X-ray magnetic circular dichroism (XMCD) has been exploited. XMCD measurements are performed at the *L*_2_ and *L*_3_ absorption edges of the constituent elements of the Heusler alloys, which represent the X-ray-induced excitation from the 2*p*_1/2_ and 2*p*_3/2_ core levels into the valence *d* states, respectively [180]. A magnetic field is applied perpendicular to the sample films, realising the magnetisation of the samples to be aligned parallel (or antiparallel) to the incident circularly polarised X-rays. These two configurations provide the corresponding X-ray absorption spectra, both of which are measured by the total electron yield method, revealing the difference in the population between up and down spin electrons. The difference in absorption cross-sections represents the XMCD signals as a result. Since the orbital part of the atomic wavefunction interacts with the circularly polarised X-rays [181], which indirectly interact with the spins of the atoms through the spin–orbit interaction [182], non-zero XMCD signals can be observed in the vicinity of the *L*_2_ and *L*_3_ edges. By applying the sum rules [181–183] after relevant background subtraction, element specific spin magnetic moments per atom *m*_spin_ are estimated as listed in Table 4.

**Table 4.** List of element-specific magnetic moments per atom for both half- and full-Heusler alloys

Heusler alloys		Total magnetic moment [μ_B_/f.u.]	X [μ_B_/atom]		Y [μ_B_/atom]		Refs.
			*m*_spin_	*m*_orb_	*m*_spin_	*M*_orb_	
NiMnSb	Exp. (f)	3.9 ± 0.2	0.2		3.0		epi-*C*1_b_-MBE film/V seed/MgO(001) sub. [84]
	*Calc.*	*3.991*	*0.245*	*0.015*	*3.720*	*0.027*	[88]
Co_2_MnGe	Exp. (b)	4.93 (RT)	~ 0.05	0.70	3.40	~ 0.03	poly-bulk [125]
	Exp. (b)	5.004 (RT)	0.975		~ 0.044		*L*2_1_-bulk [184]
			1.04		2.44		[185]
Co_2_MnGa	Exp. (b)	3.01	0.52				*L*2_1_-bulk [45]
	Exp. (b)	3.2					*L*2_1_-poly-bulk [186]
	Exp. (b)	2.5					Poly (50 nm grains) [187]
	Exp. (f)		0.534 ± 0.050		0.175 ± 0.016		(1.5 ~ 1.7) nm Al/5.3 nm epi-*L*2_1_-MBE-Co_1.95_Mn_0.98_Ga_1_/GaAs(001), Co_2_MnGa/GaAs interface [188]
			0.470 ± 0.051		0.34 ± 0.036		(1.5 ~ 1.7) nm Al/9.7 nm epi-*L*2_1_-MBE-Co_1.95_Mn_0.98_Ga_1_/GaAs(001), Co_2_MnGa bulk [189]
	*Calc.*		*2.91*		*0.65*		[189]
Co_2_MnSi	Exp. (f)	4.7 (10 K)	1.20 ± 0.05		~ 2.6		[84]
		5.1 (RT)	1.20 ± 0.05	0.10 ± 0.02	~ 2.6	0.04 ± 0.02	epi-*L*2_1_-PLD film/GaAs(001) sub. [119]
		4.7 (10 K)	1.1		1.7		1.4 nm Al-O/100 nm (011)-textured-sputtered Co_2_MnSi/Vbuffer/(SiO_2_)/Si sub. [190]
		4.8 (4 K)	1.07	0.04	2.46	0.05	epi-*L*2_1_-sputtered film/MgO(001) sub. [149]
		4.8 (4 K)	1.13	0.14	2.47	0.10	epi-*L*2_1_-sputtered film/Al_2_O_3_(11–20) sub. [149]
	*Calc.*	*5.008*	*0.994*	*0.029*	*3.022*	*0.017*	[88]
			*1.021*		*2.971*		[67]
		*4.998 ~ 5.000*	*1.08 ~ 1.158*		*2.725 ~ 3.096*		[191]
Co_2_Cr_0.6_Fe_0.4_Al	Exp. (b)	3.49 (5 K)	0.96	0.12	Cr: 0.40	Cr: 0.04	*L*2_1_-bulk [138,139]
					Fe: 2.37	Fe: 0.33	
			0.86	0.04	Cr: 0.4	Cr: 0.0	*L*2_1_-bulk [192]
					Fe: 2.17	Fe: 0.09	
		3.4 (5 K)	~ 1.2	~ 0.12	Cr: ~ 0.4	Cr: ~ 0.035	*L*2_1_-bulk [144]
					Fe: ~ 2.6	Fe: ~ 0.18	
	Exp. (f)	2.26 (RT)	1.09 ± 0.11	0.038 ± 0.004	Cr: -	Cr: -	3 nm MgO/20 nm epi-*L*2_1_-MBE film/GaAs(001) sub. [38]
					Fe: -	Fe: -	
			0.72	0.09	Cr: 0.2	Cr: 0.04	epi-*L*2_1_-sputtered film/Fe buffer/MgO(001) sub. [192]
					Fe: 1.90	Fe: 0.10	
			0.74	0.08	Cr: 0.1	Cr: 0.01	epi-*L*2_1_-sputtered film/Al_2_O_3_(11–20) sub. [192]
					Fe: 1.90	Fe: 0.10	
	*Calc.*		*0.96*		Cr: *1.52*		*L*2_1_-bulk [192,196]
					Fe: *2.77*		
		*3.56*	*0.86*	*0.042*	Cr: *1.47*	Cr: *0.005*	[144]
					Fe: *2.59*	Fe: *0.082*	
Co_2_Cr_0.625_Fe_0.375_Al	*Calc.*	*3.68*	*0.764 ~ 0.0923*	*0.021 ~ 0.048*	Cr: *1.244* ~ 0.1537	Cr: *0.001* ~ 0.0010	[128]
					F_e_: *2.469 *~ 2.787	Fe: *0.028 *~ 0.083	
		*3.8*					[193]
	Exp.	1.4 ~ 2.1					[193]
Co_2_FeAl	Exp. (f)	4.8 (5 K)	0.91 ± 0.04	0.089 ± 0.003	1.29 ± 0.05	0.089 ± 0.005	4 nm SiO_2_/20 nm epi-*L*2_1_-MBE film/GaAs(001) sub. [130,135]
			1.21	0.16	1.83	0.24	Thin film on MgO(001) [194]
		4.254	0.75	0.042	2.70	0.070	Thin film on MgO(001) [195]
	*Calc.*	*4.996*	*1.094*	*0.045*	*2.753*	*0.060*	[196]
Co_2_FeSi	Exp. (b)	5.97 ± 0.05	1.2 ± 0.1		2.0 ± 0.1		poly-*L*2_1_-bulk [158]
		5.33					Bulk [197]
	Exp. (f)		1.25	0.12	2.5	0.1	70 nm epi-*L*2_1_-sputtered Co_2_FeSi(110)/Al_2_O_3_(11–20) sub. [196]
		56.2 ± 0.5					Co_2_FeSi/GaAs(001) [154]
	*Calc.*	*6.00*	*1.54*		*3.30*		[144]
Co_2_TiSn (bulk)	Exp. (b)	1.92	0.87 ± 0.02	0.09 ± 0.02			poly-*L*2_1_-bulk [158]
	*Calc.*	*1.68*	*0.90*				[158]
Co_2_ZrSn (bulk)	Exp. (b)	1.64	0.70 ± 0.01	0.012 ± 0.01			poly-*L*2_1_-bulk [158]
	*Calc.*	*1.64*	*0.88*				[158]
Co_2_NbSn (bulk)	Exp. (b)	0.94	0.38 ± 0.01	0.09 ± 0.01			poly-*L*2_1_-bulk [158]
	*Calc.*	*1.08*	*0.43*				[158]

#### (i) Bulk properties

For NiMnSb [84], Co_2_MnGa [124], Co_2_MnSi [149] and Co_2_FeSi [196], *m*_spin_ for both Co and Y atoms show good agreement with theoretical calculations as listed in Table 4. On the contrary, for Co_2_(Cr,Fe)Al, *m*_spin_ for Co maintains the good agreement, while that for Y (Cr and Fe) decreases significantly as compared with the calculations. For the latter cases, the enhancement in *m*_orb_ is reported in general. It should be emphasised that the *m*_orb_ for Co in the epitaxial films is observed to be twice as large as the calculation [130]. Similar enhancement in *m*_orb_ for Co has also been reported in a Co_2_MnGe bulk sample [125]. Such enhancement in *m*_orb_ for the transition metals X and Y suggests that the spin-orbit coupling in the Heusler alloys are very strong and may be the main reason to induce the half-metallicity.

#### (ii) Heusler alloy/tunnel barrier interfaces

Because the XMCD measurement is sensitive to the surface of the sample, typically probing within 10 nm from the surface, the measurement always suffers from the overlap of the surface signals with the bulk signals. For films, the asymmetry and dislocation in the vicinity of the interfaces between the Heusler alloy layers and the capping layers, which are usually deposited to prevent oxidation, reduce *m*_spin_. The samples can be capped with 3 ~ 4 nm oxide layers to mimic the interface between a FM Heusler alloy layer and an oxide tunnelling barrier. In 20 nm Co_2_Cr_0.6_Fe_0.4_Al with a 3 nm MgO epitaxial capping layer, the X-ray absorption spectroscopy (XAS) spectra for both Cr and Fe possess minor splits in the peaks, corresponding to the oxidation of these elements [130]. For Co, on the other hand, no peak splitting is observed and the spin moment per atom is estimated to be 1.09 μ_B_, which almost agrees with the calculated value (0.96 μ_B_) [138]. For 20 nm Co_2_FeAl with a 4 nm cap, the spin moment per Co atom also show more than 80% of the calculated value, while that of Fe only satisfy less than 50% of the calculation [135]. These results suggest that the epitaxial *L*2_1_ Co_2_Cr_1-*x*_Fe*_x_*Al films suffer from element selective oxidation at the interface with an oxide tunnel barrier and selective atomic disorder for Fe and Al, resulting in the decrease in the spin magnetic moments for Cr and Fe.

An epitaxial *L*2_1_ Co_2_Cr_0.6_Fe_0.4_Al film has been prepared onto Al_2_O_3_(11–20) and MgO(001) substrates with an Fe buffer layer by sputtering [192]. For both cases, the spin moment for Co only shows about 15% decrease as compared with the theoretical calculations [144], while that for Cr and Fe shows almost 90% and 30% decrease. This decrease is also attributed to the atomic disorder between Co and Cr/Fe atoms.

Elmers et al. have reported the orbital magnetic moment per spin, *r* = *m*_orb_/(*m*_spin_ + *m*_dipole_), to be (0.14 ± 0.02) for Co and (0.06 ± 0.02) for Fe in the Co_2_FeAl bulk samples [138,139]. For the *L*2_1_ epitaxial Co_2_FeAl films, by neglecting the magnetic dipole term, *m*_dipole_, *r* is estimated to be (0.098 ± 0.007) and (0.069 ± 0.005) for Co and Fe, respectively [135]. These values imply that Co does not show any enhancement in *m*_orb_, while Fe shows similar enhancement as the bulk. Even so, it should be emphasised that *m*_orb_ for Co in the epitaxial films is observed to be twice as large as the calculation (see Table 4). Similar enhancement in *m*_orb_ for Co has been reported in a Co_2_MnGe bulk sample [125]. Such enhancement in *m*_orb_ for the transition metals X and Y suggests that the spin-orbit coupling in the Heusler alloys are very strong and maybe the main reason to induce the half-metallicity. For sputtered Co_2_MnAl film with the atomically disordered *B*2 structure, the Gilbert damping constant is found to be small by ferromagnetic resonance (FMR) measurement (see more details in Sec. 4.8), indeed indicating weak spin–orbit interaction for the disordered phases [113]. The large spin–orbit interaction in the Heusler alloys has also been suggested from a large AMR effect observed in polycrystalline Co_2_MnGa film, as large as 6% at RT (8% at 1.6 K) [124].

#### (iii) Heusler alloy/substrate interfaces

Total (spin and orbital) moments of *L*2_1_ Co_2_MnGa films epitaxially grown on GaAs(001) by MBE has been systematically investigated to specifically observe the difference between the bulk and interface regions [189]. They have maintained almost the same thickness for their capping layers (1.5 ~ 1.7 nm) for these samples with changing the Heusler layer thickness to be 5.3, 7.6 and 9.7 nm. For the first sample, they observe that the total moments for Co are less than 20% of the calculation (and also observation for bulk), while that for Mn is less than one-third of the calculation and bulk. For the last sample, on the other hand, they find slight recovery in the Mn total moment up to over 50% of the calculation and bulk. A similar tendency has also been found in off-stoichiometric samples. These results strongly suggest the presence of a magnetic dead layer in the Heusler alloy films near the GaAs interface.

Epitaxial *L*2_1_ sputtered Co_2_FeSi(110) on an Al_2_O_3_(11–20) substrate has been investigated by XMCD to reveal the thickness dependence of the element-specific magnetic moments [192]. They observe approximately 80% of the calculated spin moments for both Co and Fe atoms with the film thickness larger than 10 nm. In the vicinity of the Al_2_O_3_ interface, a 0.8 nm thick magnetic dead layer is also found to be formed at RT.

### 4.5. Andreev reflection

Soulen et al. have first applied Andreev reflection to measure the spin-polarisation *P* of FM materials [36], and afterwards, this technique has been widely used to measure *P* of the Heusler alloys as listed in Tables 1 and Tables 2. This technique is based on the spectroscopic measurement in an FM/insulator/superconductor tunnelling junction developed by Tedrow and Meservey [198]. For the Andreev reflection, a superconducting point contact is used instead of a superconducting film, which allows to achieve spin-polarised electron injection into an FM film with forming a coherent pair with an oppositely spin-polarised electron in FM, while reflecting an Andreev hole back to the superconductor. This process occurs in addition to the conventional ohmic response at the interface. HMF with *P* = 100%, however, cannot offer the coherent pair when the majority spin is injected from the point contact due to the absence of the minority spins at *E*_F_. Although this is a very powerful technique to measure *P* directly, the estimated *P* typically reflects the spin DOS in the vicinity of the surface.

Shigeta et al. have reported the spin polarisation of Ru_2-*x*_Fe*_x_*CrSi Heusler alloys using the Andreev reflection [199]. Apart from determination of the spin polarisation, it also shows the structural ordering of RuFeCrSi depends on the Fe and Ru content. For 0.1 ≤ *x* < 1.8, the *L*2_1_ ordering is confirmed, whilst for *x* = 1.8, the crystalline structure becomes *B*2 [200,201]. The spin polarisation of *x* = 1.5 and 1.7 is measured to be *P* = 53 and 52%, respectively. Shigeta et al. also determined the spin polarisation of Co_2_FeSi, which is integrated with superconductor NbN on a MgO substrate. The average spin polarisation of Co_2_FeSi is reported to be (52 ± 2)%. Similar study on the other Co-based Heusler alloy, bulk Co_2_FeGa with the *L*2_1_ ordering, is reported by Zhang et al. [178]. XRD measurement is undertaken for the bulk sample. The spin polarisation is measured to be *P* = 59%. Recent study has also reported spin polarisation of (54 ± 2)% at clean Co_2_FeSi/Nb interface in CPP-GMR-type junctions [202].

### 4.6. Bandgap measurements

Resolving the true bandgap of the half-metallic Heusler alloys was long a significant challenge due to the spin-dependent nature of the gap. The gap could only be estimated by Andreev reflection (see Section 4.5), which are limited by the interfacial quality. Alhuwaymel et al. successfully showed a new technique for determining the spin bandgap using circularly polarised infrared (IR) radiation [68]. By sweeping the IR energy spectrum with an applied magnetic field both anti- and parallel to the polarisation of the radiation, the minority and majority bands can be probed independently. An absorption peak will be observed in only one orientation as the spin-polarised electrons which were excited by this radiation were absorbed by the bandgap of the Heusler alloy, in this case the full-Heusler alloys Co_2_FeSi [68] and Co_2_FeAl_0.5_Si_0.5_ [203], with values of ~94 and ~110 meV were found, respectively.

Similarly, light absorption spectroscopy has been used by Schmitt et al. on thermoelectric materials such as ZrNiSn half-Heusler alloys [204]. By varying the composition, such as via Sc-doping the semi-conducting nature of the minatory band can be switched from *p*- to *n*-type and the resulting bandgap observed. This highlighted several discrepancies with *ab initio* calculations due to hole/electron contributions, where a value of 0.13 eV compared to the theoretical value of 0.5 eV. These discrepancies are also in observed in the method of Alhuwaymel et al., as well as the work of Aliev et al. [205], suggesting that there is much to be done in the true determination of half-metallic bandgaps.

### 4.7. Gilbert damping constant

Gilbert damping constant *α* is the friction coefficient of magnetisation motion, which affects the switching speed of magnetisation reversal process. From a technological point of view, small *α* is required to reduce the switching current density *J*_c_ for the current-induced magnetisation switching process, because *J*_c_ is proportional to *α* [206]. According to the torque-correlation model proposed by Kambersky [207], *α* is proportional to the density of states, DOS, at *E*_F_. Therefore, it is expected that half-metallic materials which have an energy gap in one spin channel exhibit relatively small *α*. In experiments, *α* can be evaluated using ferromagnetic resonance (FMR) [208], or time-resolved magneto-optical Kerr effect (TR-MOKE) [209]. Table 5 is a summary of reported values of *α* in experiments for Heusler alloys. Many of those presented relatively small *α* of less than 0.01. Mizukami et al. discussed correlation between the calculated DOS and the experimentally determined relaxation frequency *G* (= *γαM*_s_, where *γ* and *M*_s_ are gyromagnetic ratio and saturation magnetisation, respectively) for Co_2_FeAl films with changing the degree of order for the *B*2 phase, *S*_B2_: *G* and the number of DOS at *E*_F_ exhibited similar trend on *S*_B2_ suggesting that the Gilbert damping is small half-metallic materials for which DOS is small at the *E*_F_ [210]. Similar discussion has been done for other compositions such as CoFeGe_0.5_ [211], Co_2_(Fe,Mn)Si [212], Co_2_Mn(Al,Si) [212], Co_2_MnSi [213], CoFeMnSi [214] and so on. Recently, quite small *α* of less than 0.001 has been experimentally demonstrated for Co_2_MnSi films [213,215], which is done by improving the degree of *L*2_1_ order possibly resulting in the improvement of the half-metallic property. The small *α* of the Heusler alloy films is suitable for reducing *J*_c_ of the current-induced magnetisation switching phenomena as well as for applying to a channel layer of spin-wave propagation experiments [216,217].

**Table 5.** List of Gilbert damping constant *α* reported for Heusler alloy films

Material	Structure	Thickness (nm)	*α*	Method	Reference
Co_2_MnSi	*L*2_1_	30	0.005	FMR	[212,218]
		5	0.03		
		30	0.002	TR-MOKE	[219]
Co_1.9_Mn_1.1_Si	*L*2_1_	30	0.0007	FMR	[213]
		10	0.0007	FMR	[215]
Co_2_FeSi	*L*2_1_	30	0.022	FMR	[212,218]
		3	0.02		[220]
		20	0.0018	FMR	[221]
Co_1.75_Fe_1.25_Si	*L*2_1_	20	0.0012	FMR	[221]
Fe_2_CoSi	*L*2_1_	20	0.0019	FMR	[221]
Co_2_Fe_0.4_Mn_0.6_Si	*L*2_1_	30	0.004	FMR	[212,218]
		3	0.013		[220]
CoFeGe_0.5_	*B*2	50	0.0025	FMR	[211]
Co_2_FeAl	*L*2_1_	50	0.001	FMR	[210]
		140	0.005	FMR	[222]
	*B*2	50	0.003	FMR	[223]
	*B*2 and *A*2	1	~0.015	TR-MOKE	[224]
Ni_2_MnSn	*L*2_1_	40	0.0075	FMR	[225]
CoFeMnSi	*L*2_1_	10	0.0027	FMR	[214]

### 4.8. Nuclear magnetic resonance

Nuclear magnetic resonance (NMR) is based on the interaction between the spin of a nucleus with the surrounding fields, such as external magnetic field, neighbouring nuclei, surrounding electrons of the nucleus, and surrounding electrons of neighbouring atoms [226,227]. NMR is a good probe to characterise the local information of Heusler alloys. Because the shape of resonance spectrum is sensitive to the change of hyperfine field reflecting the local environment. Chemical degree of order as well as the chemical composition can be quantitatively evaluated using NMR: *e.g*., Wurmehl et al. carried out an NMR study on Co_2_Mn_1-*x*_Fe*_x_*Si Heusler alloys with several Fe composition *x* in the bulk samples, and quantitatively evaluated the chemical ordering especially focusing on the distribution of Fe-Mn atoms using spin-echo NMR of ^55^Mn nuclei [228]. The chemical ordering between Fe and Mn is not easy to characterise using, *e.g*., laboratory X-rays because the atomic numbers of those are close each other, thus NMR is useful for characterising Heusler alloys which often consists of atoms with similar atomic numbers. For further detail of NMR study on Heusler alloys, review articles are found in refs. [229,230].

### 4.9. Mössbauer spectroscopy

Mössbauer spectroscopy is a spectroscopic technique on resonant absorption of *γ*-ray by nuclei in solids, which provides information of local electronic states of the material. Experimental studies on Heusler alloys using Mössbauer spectroscopy have been reported since 1960’s, in which bulk samples containing Sn or Fe, *e.g*., Co_2_MnSn [231], Fe_3_Sn [232], were selected, because ^57^Fe and ^119^Sn isotopes are suitable for NMR measurements in laboratories. Heusler alloys of films forms have been also studied using Mössbauer spectroscopy in the last decade, which revealed local magnetic properties of ternary- and quarterly-Heusler alloy films quantitatively [233–235]. Mössbauer spectroscopy can be also applied for studies of interfaces in layered film samples, which is useful for optimisation of spintronic devices, because magnetic properties at the interface sensitively affect the spin-dependent transport in MTJs or CPP-GMR junctions. Tanaka et al. fabricated layered film samples consisting of a MgO/Co_2_FeGe structure for MTJ application, using molecular beam epitaxy technique. They controlled the metallic termination layer at the interface: Co-termination or ^57^Fe-Ge termination, and discussed hyperfine-field distribution at the ^57^Fe sites [236]. Similar study has also been done for metallic-layered films consisting of a Co_2_FeGe/Ag structure for CPP-GMR junctions. The dependence on the deposition temperature of Co_2_FeGe layer was discussed for the degree of order at the Co_2_FeGe/Ag interface, which is consistent with the CPP-GMR ratios using similar sample structures [237]. Review articles of Refs. [230,238] are to be referred for further detailed studies on Heusler alloys using Mössbauer spectroscopy.

### 5. Applications of ferromagnetic Heusler alloys

#### 5.1. Spin injection

In order to achieve highly efficient spin injection from an FM film into a semiconductor or an NM metal, two distinct approaches have been proposed theoretically; spin injection from FM with almost 100% spin polarisation, such as HMF and DMS, in a diffusive regime [19], and that through a tunnel barrier in a ballistic regime [239]. For the former case, the Heusler alloy is one of the best candidates due to their good lattice matching with major semiconductors and their high *T*_C_ as discussed above. Diffusive spin injection holds a key to realise the spin-polarised three-terminal devices, such as a spin-polarised field-effect transistor [240] and a lateral spin-valve [241] at RT.

A NiMnSb(111)/CdS(111) interface is suggested to suppress the spin-flip electron transport at *E*_F_ [94], indicating the possible use of the NiMnSb film as a spin-filter to inject a spin-polarised electron current into the semiconductor. A similar effect is expected even for the electron transport in the (110) orientation in a non-half-metallic Ni_2_MnIn/InAs interface, resulting in *P* ~ 80% [168]. At this interface, only the electron spins within an energy of *k*_B_*T* ≈ 1/40 eV at *E*_F_ in Ni_2_MnIn are travelled into InAs. *E*_F_ must be close to the conduction band of InAs for the spins to fill these unoccupied states. Since the conduction minimum occurs at the Γ point, the transmittances for the minority spins are calculated to depend on the crystalline directions; 0.75, 0.82 and 0.99 for the [100], [110] and [111] directions, respectively. For the majority spins, on the other hand, the transmittances are calculated to be 0.19, 0.19 and 0.39 for the corresponding directions, respectively, since large spin-orbit scattering is expected at the interface to change the in-plane momentum to match an available state in InAs.

For the case of the Co_2_CrAl/III–V semiconductor interfaces, the half-metallicity is calculated to be preserved for certain interfacial combinations. For Co_2_CrAl/GaAs interfaces, the half-metallicity is preserved for the Co/As interface on the GaAs(001) surface and for Al/As on GaAs(110), while it is demolished for CrAl/As on GaAs(001) [132]. For Co_2_CrAl/InP interfaces, the Cr spin moment is calculated to be enhanced for both the CrAl/In and CrAl/P interfaces (*P* ~ 63% and 65%, respectively), while it is decreased for both the Co/In and Co/P interfaces (*P* ~ 56% and ~ 74%, respectively), even though the Cr spin moment is almost the same as the bulk value for both cases [133].

Experimentally, spin injection has been reported in a Co_2.4_Mn_1.6_Ga/InGaAs quantum well (QW) structure [123]. Although *P* is measured to be ~50% by the Andreev reflection, the injected electron spin polarisation is estimated to be 13% at 5 K, which is smaller than that for an Fe/InAs QW. It is therefore essential to fabricate a sharp Heusler alloy/semiconductor interface with a relevant band matching as theoretically proposed in order to achieve highly efficient spin injection.

In a lateral spin-valve structure, Co_2_FeSi has been used as an FM injector into Cu, achieving 27% spin injection efficiency [242]. This is the highest efficiency reported to date based on the optimised fabrication technique.

### 5.2. Anisotropic magnetoresistance effect

A recent theoretical work predicts that a sign of an anisotropic magnetoresistance (AMR) ratio of half-metallic materials is negative [243]. An AMR ratio effect has been systematically investigated in Co_2_MnZ and Co_2_FeZ (Z = Al, Si, Ge and Ga) epitaxial films [244]. The sign of an AMR ratio is negative when the total valence electron number is between 28.2 and 30.3, while that is positive when the total valence electron number is below 28.2 and above 30.3. These findings indicate that *E*_F_ overlaps with the valence or conduction band edges of half-metallic gap with the valence electron number to be either ∼ 28.2 or 30.3, respectively. Half-Heusler NiMnSb epitaxial films also show a negative AMR ratio [245,246]. Recently, a further study has been achieved by Sato et al. on epitaxial Co*_x_*(Mn_0.44_Ga_0.56_)_100-*x*_ thin films. The Co composition of *x* was controlled between 47.4 and 52.6 at%. The corresponding AMR ratios were measured by changing the electric current relative to the magnetic field directions between 5 and 300 K. The results show that the sign of the AMR ratios changes according to the current directions. The maximum AMR ratio is obtained at *x* = 49.7 at% [247]. AMR can be used as a way to optimise a composition of half-metallic Heusler alloys for the RT half-metallicity. The magnitude of the negative AMR ratio gradually increases with shifting *E*_F_ away from the gap edges.

### 5.3. Giant magnetoresistive junctions

#### 5.3.1. In-plane magnetic anisotropy

##### (i) Half-Heusler alloys

GMR junctions with FM Heusler alloy films have been studied over the last decades to achieve the requirement for the next-generation magnetic storage and memory [248] as shown in Figure 1. Both current-in-plane (CIP) and current-perpendicular-to-the-plane (CPP) GMR have been investigated with [NiMnSb/Cu]_10_, [NiMnSb/Cu]_10_, NiMnSb/Cu/Ni_0.80_Fe_0.20_ (or NiMnSb/FeMn) and NiMnSb/Cu/Ni_0.80_Fe_0.20_ (or NiMnSb/FeMn) [249,250]. The former multilayers show a CPP-GMR ratio of 4.5% at 4.2 K. The latter spin-valves show a CPP-GMR ratio of 7.2% at 4.2 K.

Similar progress can be found for the GMR junctions with the half-Heusler alloy films. PtMnSb films are deposited on Al_2_O_3_(0001) by sputtering to form a spin-valve structure, PtMnSb(111)/CuMnSb(111)/PtMnSb(111)/MnFe, showing 0.47% GMR at RT [96]. This small GMR ratio may be due to the empty site disorder and interfacial defects. As discussed in Sec. 3.1, the terminations and crystalline directions of the Heusler alloy films can control the effective spin polarisation, which needs to be optimised in a junction. Recently, a CPP-GMR ratio of 8% at RT (21% at 4 K) has been reported in fully epitaxial NiMnSb (20)/Ag (5)/NiMnSb (7) (thickness in nm) junctions with the (001) orientation [246]. The junctions achieve *RA* = (26 ± 1)×10^−3^ Ω·μm^2^. By repeating two sets of epitaxial GMR junctions, consisting of NiMnSb (9)/Ag (5)/NiMnSb (3)/Ag (5)/NiMnSb (9) (thickness in nm), further increase in the CPP-GMR ratio up to 11% (41% at 4 K) has been reported by using a dual spacer [251]. Here, *RA* is found to be reduced to 3.9 × 10^−3^ Ω·μm^2^, which is favourable for device applications as mentioned above.

##### (ii) Full-Heusler alloys

In 2001, a GMR junction, consisting of Co_2_MnGe (6)/V (1.6)/Co_2_MnGe (3)/Fe (0.3)/ZnSe (50)/GaAs(001) (thickness in nm) have been fabricated and measured along the two [110] directions [252]. The corresponding GMR ratio is measured to be less than 1%. Since then, a series of GMR junctions have been designed and evaluated, especially utilising an epitaxial film to achieve high crystalline ordering. Epitaxial films are typically deposited on a MgO(001) substrate with the crystalline relationship Co2Cr0.6Fe0.4Al(001)[100]||MgO(001)[110] or a Ag seed layer. For a CIP configuration, a multilayer of a [Co_2_Cr_0.6_Fe_0.4_Al (10)/Cu (2.5)/Fe_0.1_Co_0.9_ (8.1)] stack (thickness in nm) has been used with showing only 2% GMR at RT (4% at 15 K) [129]. Co-Fe-Al has then been used to show up to 3.3% CPP-GMR ratio at RT [253,254], followed by Co_2_MnSi with a Cr spacer with the CPP-GMR ratio of 2.4% and *RA* of 19 × 10^−3^ Ω·μm^2^ [255]. By replacing the Cr spacer with Cu to minimise the interfacial atomic diffusion and mixing, the CPP-GMR ratio is increased to 8.6% at RT (30.7% at 6 K) [256]. In parallel, the Ag spacer has been commonly used, achieving a CPP-GMR ratio of 6.9 (14)% at RT (6 K) using Co_2_FeAl_0.5_Si_0.5_/Ag/Co_2_FeAl_0.5_Si_0.5_ [257]. Further enhancement has been reported for a CPP configuration, *e.g*., Co_2_MnSi (8.8)/Ag (5)/Co_2_MnSi (8.8) (thickness in nm) with a GMR ratio and *RA* of 28.8% and 3.1 × 10^−2^ Ω·μm^2^ at RT, respectively [258], and Co_2_FeAl_0.5_Si_0.5_ (2.5)/Ag (5)/Co_2_FeAl_0.5_Si_0.5_ (2.5) (thickness in nm) with a GMR ratio and *RA* to be 34% (80% at 14 K) and 1.5 × 10^−2^ Ω·μm^2^ at 290 K, respectively [259].

In 2011, a large CPP-GMR ratio of 42% has been reported using Co_2_FeGe_0.5_Ga_0.5_/Ag/Co_2_FeGe_0.5_Ga_0.5_ junctions [260], followed by the increase to 59.6 (200)% at RT (10 K) with a AgZn spacer [261]. Theoretically, a larger GMR ratios are expected, *e.g*., 90% and ~60% for *L*2_1_- and *B*2-Co_2_MnAl/Ag/Co_2_MnAl junctions, respectively [262]. A Co_2_Fe_0:4_Mn_0:6_Si/Ag/Co_2_Fe_0:4_Mn_0:6_Si junction with the *L*2_1_ ordering shows further increase in the CPP-GMR ratio of 74.8% with *RA* of (67.6 ~ 369.2)×10^−3^ Ω·μm^2^. Co_2_Fe_0.4_Mn_0.6_Si/Ag/Co_2_Fe_0.4_Mn_0.6_Si shows a 58 (184)% GMR ratio at RT (30 K) [263,264]. Further improvement has been achieved by sandwiching the Ag spacer with NiAl as Co_2_Fe(Ga_0.5_Ge_0.5_)/NiAl/Ag/NiAl/Co_2_Fe(Ga_0.5_Ge_0.5_), showing the GMR ratio of 82 (285)% at RT (10 K) and *RA* of 4 × 10^−2^ Ω·μm^2^ [265]. This is the largest GMR ratio reported to date, indicating that further improvement in the GMR should be achievable by controlling the interfacial smoothness without atomic defects in the junction to meet the roadmap produced by the IEEE Magnetics Society [39].

##### (iii) Alternative spacers

A *L*1_2_ Ag_3_Mg spacer has been used in a CPP-GMR junction with epitaxial Co_2_Fe_0.4_Mn_0.6_Si Heusler alloy films with <3% lattice matching [266]. The maximum RA and the GMR ratio are reported to be 25 × 10^−3^ Ω·μm^2^ and 63%, respectively, at RT. A review on CPP-GMR junctions with Heusler alloys can be found in Ref [267]. The counterpart approach from MTJ is described in Section 5.4.1(iii). Further reduction in *RA* has been achieved with In-Zn-O and 0.2 ~ 0.6 nm thick Ag insertion, which is similar to nano-oxide layer (NOL) structures demonstrated by Toshiba [268], showing *RA* ~ 0.1 Ω·µm^2^ and an MR ratio of 32% [269].

### 5.3.2. Perpendicular magnetic anisotropy

Since the discovery of the perpendicular magnetic anisotropy (PMA) at an Fe/MgO interface [16,270–271], MgO layer insertion has been widely used to induce PMA, which is, however, not applicable for a GMR junction. Mn-based binary Heusler alloys are alternative choices [272]. Recently, a body-centred cubic (bcc) seed layer has been used to minimise the interfacial mixing with fcc Heusler alloy layer [273]. For a bcc vanadium seed layer, X-ray analysis shows that 25-nm-thick vanadium introduces a strong (110) orientation in the Co_2_FeSi Heusler alloy. The *B*2-texture of the Co_2_FeSi is found to match that of the vanadium proving that the texture is defined by the seed layer. Reduction of the Co_2_FeSi thickness is found to result in a reduction in the strength of the in-plane anisotropy, as expected from the cubic nature. Since the perpendicular magnetic anisotropy (PMA) is induced at the interface between Co_2_FeSi and V, a second vanadium interface is added and found to increase the observed PMA. Further reduction in the thickness of the Co_2_FeSi layer lead to an increase in the PMA where 4-nm-thick Co_2_FeSi exhibited a strong PMA (see Table 6).

**Table 6.** List of measured saturation magnetisation (*M*_S_) and perpendicular magnetic anisotropy (PMA) for major Heusler alloys

Heusler Alloys	*M*_S_ (emu/cm^3^)	PMA (erg/cm^3^)	Refs.
Mn_3_Ga	500	1.5 × 10^7^	[274]
Mn_2.5_Ga	250	1.2 × 10^7^	[275]
Co_2_FeAl/MgO	1000	(2 ~ 3)×10^6^	[276]
	1100	3.1 × 10^6^	[277]
	731	1.9 × 10^6^	[278]
Co_2_FeAl/MgAl_2_O_4_	1200	4.2 × 10^6^	[279]
V/Co_2_FeSi	700	1.75 × 10^3^	[273]
W/WO_x_/Co_2_FeSi	400	4.00 × 10^3^	[280]
W/Co_2_FeSi	600	–	[281]

For further improvement in PMA, tungsten is selected due to the lower bulk resistivity of 5.6 × 10^−6^ Ω·cm [282], which is almost a half of that of vanadium (1.9 × 10^−5^ Ω·cm) [283]. High-temperature growth at 673 K is applied for tungsten, providing the (110) surface orientation as well [280,281]. The magnetisation of the W/Co_2_FeSi sample is measured to be 400 emu/cm^3^ with the perpendicular anisotropy of 8 × 10^5^ erg/cm^3^ as summarised in Table 6. Since the crystalline plane induced by the bcc seed layers is (110), which is a favourable orientation to promote the layer-by-layer crystallisation, low-temperature crystallisation has been demonstrated with PMA [281]. Samples consisting of W (10)/Co_2_FeAl_0.5_Si_0.5_ (12.5)/W (1.2)/Co_2_Fe Al_0.5_Si_0.5_ (2.5)/Ta (2) (thickness in nm) have been deposited at 355 K for 2 min. *M*_S_ reaches ~1060 emu/cm^3^, which is almost 85% of the theoretically predicted value, and it is ideal for device implementation due to the low-temperature crystallisation. A GMR junction with an Ag spacer unfortunately shows only 0.03% at RT, which requires further optimisation for device implementation.

### 5.4. Magnetic tunnel junctions

#### 5.4.1. In-plane magnetic anisotropy

**Figure 11.**
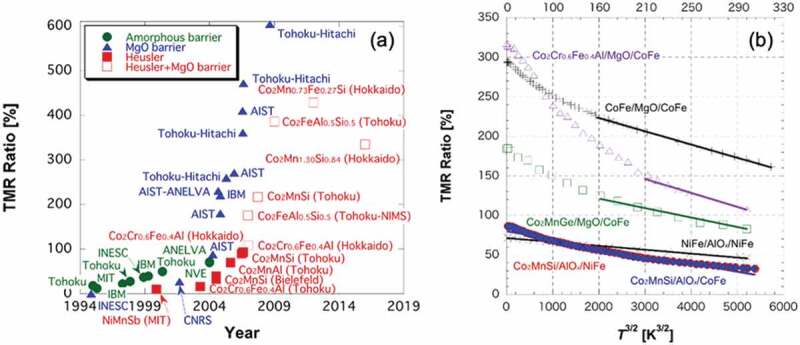
(a) Recent progress in TMR ratios with different junctions. (b) Temperature dependence of TMR ratios for MTJs with Co-based full Heusler alloy films. Experimental data are taken from Refs. [292] for Co_2_MnGe/MgO/CoFe, [293], for Co_2_Cr_0.6_Fe_0.4_Al/MgO/CoFe, [118] for Co2MnSi/Al-O/CoFe, [148] for Co2MnSi/Al-O/NiFe, [294] for Co_2_MnSi/Al-O/CoFe and Co2MnSi/MgO/CoFe, [290] for Co_2_FeAl_0.5_Si_0.5_/MgO/Co_2_FeAl_0.5_Si_0.5_ and [295] for Co_2_Mn_1.30_Si_0.84_/MgO/Co_2_Mn_1.30_Si_0.84_ as well as NiFe/Al-O/NiFe, [14] for CoFe/MgO/CoFe and [296] for CoFeB/MgO/CoFeB

As shown in Figure 11(a), TMR ratios have not been increased over 10 years since the report by Ikeda et al. with coherent tunnelling using a MgO barrier [16]. Great efforts have hence been devoted for the increase in the TMR ratios with half-metallic Heusler alloy films as detailed below.

***(i)*** ***Half-Heusler alloys*** An epitaxial half Heusler NiMnSb film has been first used as an electrode in MTJ, showing 9% TMR at RT [77]. Apart from this pioneering work, very few studies were performed on MTJ with half-Heusler alloys.

***(ii) Full-Heusler alloys***

*(a) Co_2_(Cr,Fe)Z* An epitaxial full Heusler Co_2_FeAl film with the *L*2_1_ structure is also applied for MTJ but shows only 9% TMR at RT [130]. The small TMR ratios may be caused by the selective oxidation at the interface between the Heusler films and the oxide barriers according to XMCD measurements [38]. Recently, an epitaxial *L*2_1_ Co_2_Cr_0.6_Fe_0.4_Al film sputtered onto a MgO(001) substrate has been adopted for a fully epitaxial MTJ with the structure of Co_2_Cr_0.6_Fe_0.4_Al/MgO/CoFe, showing 42% at RT (74% at 55 K) [140]. Even though this film possesses the epitaxial relationship Co2Cr0.6Fe0.4Al(001)[100]||MgO(001)[110], the magnetic moment is estimated to be 3.3 μ_B_/f.u., which is smaller than the calculation (3.7 μ_B_/f.u.) [141]. This indicates that the film contains an atomically disordered phase, which may also be suggested from the decrease in the TMR ratios below 55 K. Further optimisation results in the TMR ratio to become 109% at RT and 317% at 4 K with *RA* ~ 3 × 10^4^ Ω·µm^2^ [284]. The TMR ratios have further been increased to 330% at RT (700% at 10 K) with *RA* = 1 × 10^3^ Ω·µm^2^ in MTJ with Co_2_FeAl/MgO/Co_0.75_Fe_0.25_ by utilising the Δ_1_-band connection between Co_2_FeAl and MgO [285]. Using a MgAl_2_O_4_ barrier instead of MgO to maintain the Δ_1_-band connection and to make better lattice matching with *B*2-Co_2_FeAl, TMR ratios are found to be increased to 342% at RT (616% at 4 K) with *RA* = 2.5 × 10^3^ Ω·µm^2^ [286]. The departure of the TMR ratios from theoretically predicted almost infinity may also be due to the interfacial atomic disorder, due to the presence of a light element of aluminium segregated from the matrix.

For polycrystalline sputtered full Heusler MTJs, on the other hand, much smaller TMR ratios have been reported. MTJ with the structure of Co_2_Cr_0.6_Fe_0.4_Al/AlOx/CoFe shows 16% TMR at RT [73], which is later improved up to 19% at RT by the barrier optimisation [132]. By introducing a MgO underlayer, (001)-textured polycrystalline MTJs on a thermally oxidised Si substrate have been reported. MTJ with the structure of Co_2_FeAl_0.5_Si_0.5_/MgO/Co_2_FeAl_0.5_Si_0.5_ shows 125% at RT and 196% at 7 K [287]. Later, an improved TMR of 175% was reported at RT using a Co_2_FeAl/MgO/CoFe structure [288].

By replacing a half of Al with Si in Co_2_FeAl to stabilise the crystallisation, MTJs with an oriented MgO barrier for which TMR ratios of 175% have been achieved at RT when using *B*2-Co_2_FeAl_0.5_Si_0.5_ [280]. Using *L*2_1_-Co_2_FeAl_0.5_Si_0.5_, the TMR ratios of 386% at RT and 832% at 9 K with *RA* = 80 × 10^3^ Ω·µm^2^ has been reported later [290]. The decrease in the TMR ratio with increasing temperature is much faster than the temperature dependence of the magnetisation *T*^3/2^, suggesting that a small fraction of atomically disordered phases cannot be ignored in the spin-polarised electron transport at finite temperatures [see Figure 11(b)] [291]. The elimination of such disordered interfacial phases improves the TMR ratios further and realises the half-metallicity at RT.

Theoretical calculations also suggest that the interface states within the half-metallic bandgap at the half-metal/insulator interfaces prevent the highly spin-polarised electron transport [296]. This is because the tunnelling rate is slower than the spin-flip rate, and therefore the interface states for the minority spins are effectively coupled to the metallic spin reservoir of the majority spin states. In order to avoid the spin-flip scattering, a sharp interface without the interface states is crucially required. Ležaić et al. discussed that the spin polarisation of half-metallic Heusler alloys can reduce significantly at temperatures much lower than their Curie temperatures even in bulk due to the change in hybridisation by spin fluctuation [297]. The other theoretical calculations revealed that an exchange stiffness constant of an interface at Co-based Heusler alloys and a MgO barrier is significantly smaller than that of bulk [298]. Such a situation leads to serious reduction of an effective spin-polarisation at finite temperatures due to enhanced thermal fluctuation of spin moments, inducing the large temperature dependence of a TMR ratio [299]. In addition, large lattice mismatch between Co-based Heusler alloys and MgO, typically 5 ~ 7% for (001) growth, can easily introduce many misfit dislocations at the interface, which can cause suppression of the coherent tunnelling and increase the spin-flip scattering contribution [300]. Thus, introducing a larger exchange stiffness material at the interface, *e.g*., ultrathin Co-Fe [301], and a smaller lattice-constant barrier, *e.g*., MgAl_2_O_4_ [302], can be a possible solution to improve an RT TMR ratio. These recent theoretical studies suggest that the elimination of interfacial states formed by lattice mismatch and atomic mixing can minimise the temperature dependence of the TMR ratios.

*(b) Co_2_MnZ* Similarly, an MTJ with Co_2_MnAl/Al-O/CoFe shows 40% TMR at RT [110], followed by the further improvement up to 61% at RT (83% at 2 K) [131]. All of these Heusler films in the MTJs have been reported to be *B*2 structure. By comparing the TMR ratios at RT with those at low temperature, the ratios are found to show very weak temperature dependence as similarly observed for a conventional metallic MTJ as shown in Figure 11.

An MTJ with an epitaxial *L*2_1_-Co_2_MnSi film has been reported to show very large TMR ratios of 70% at RT and 159% at 2 K with *RA* = 10^6^ Ω·µm^2^ [122]. These values are the largest TMR ratios obtained in MTJ employing a Heusler alloy film and Al-O barrier. This is purely induced by the intrinsic *P* of the Heusler electrodes. These MTJs with a highly ordered Co_2_MnSi film show strong temperature dependence on the contrary to the less-ordered MTJs as discussed above; 33% at RT and 86% at 10 K [118], and 70% at RT and 159% at 2 K [122]. Such a rapid decrease in the TMR ratio with an increasing temperature is similar to that observed in MTJs with Co_2_(Cr,Fe)Al.

By replacing Al-O with MgO, a fully epitaxial MTJ, consisting of Co_2_MnSi/MgO/Co_2_MnSi, has been reported to achieve much higher TMR ratios, 217% at RT (753% at 2 K) [294] and 236% at RT (1,135% at 4 K), but with larger *RA* of 3 × 10^7^ Ω·µm^2^ [303]. Further improvements in the TMR ratio to be 354% at RT (1,995% at 4 K) have been achieved in the same system [304], followed by 366% at RT (2,110% at 4 K) with *RA* = 10^8^ Ω·µm^2^ [305]. Partial substitution of Mn with Fe in these MTJs to form Co_2_Mn_0.73_Fe_0.27_Si, TMR ratios are increased to 429% at RT (2,610% at 4 K) with *RA* = 7 × 10^7^ Ω·µm^2^ [306], which is the largest TMR ratio reported in a MTJ to date. A similar MTJ with Co_2_MnGe/MgO/Co_2_MnGe has been fabricated to show similar TMR ratios of 220% (650% at 4 K), but with large *RA* of 2.2 × 10^6^ Ω·µm^2^ [307]. The other Heusler alloys, such as CeFeMnSi, show much steeper temperature dependence of the TMR ratios, 101 and 521% at RT and 10 K, respectively [308]. The majority spin band with the ∆_1_ symmetry near *E*_F_ is calculated to be significantly modified by the Mn-Fe swapping disorder, opening an additional path for tunnelling and reducing the TMR ratio with elevating temperature. Such interfacial disorder may also be responsible for the strong temperature dependence.

*(iii) Alternative tunnelling barriers* For the *RA* reduction, alternative tunnelling barriers have been investigated. CuIn_0.8_Ga_0.2_Se_2_ semiconducting barrier with almost perfect lattice matching with Co_2_FeGa_0.5_Ge_0.5_ shows MR ratios of 40 and 100% at RT and 8 K, respectively, with *RA* of 0.3 ~ 3 Ω·µm^2^ [309]. A CuGaSe_2_ barrier shows MR ratios of 100 and 246% at RT and 30 K, respectively, with the *RA* < 1 Ω·µm^2^ [310].

### 5.4.2. Perpendicular magnetic anisotropy

#### (i) Binary Heusler alloys

**Figure 12.**
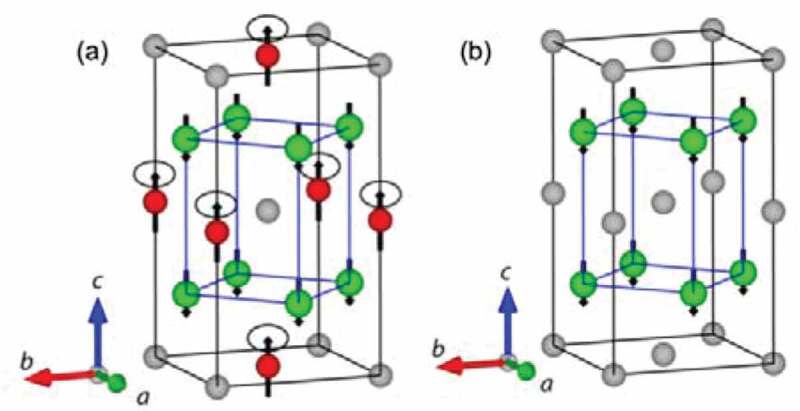
(a) The FI structure of *D*0_22_ Mn_3_Ga or *D*0_22_ Mn_3_Ge exhibiting overall *c*-axis anisotropy but with some in-plane component indicated by small circles. (b) FM structure of *L*1_0_ MnGa with Mn atoms coupled ferromagnetically [314]. Reprinted with permission from Hirohata et al. [279]. Copyright 2018. MDPI AG

By replacing Y atoms with X atoms, binary Heusler alloys can be formed. For example, Mn_3_Ga shows ferrimagnetic (FI) behaviour in the tetragonal *D*0_22_-phase with perpendicular magnetic anisotropy, as schematically shown in Figure 12(a). The FI Mn_3_Ga has been reported to possess a large uniaxial anisotropy of 1 × 10^7^ erg/cm^3^ [311] and high Curie temperature of ~770 K [312]. Mn_3_Ga has been used in an MTJ, consisting of Mn_3_Ga/MgO/CoFe and has shown 9.8% TMR at 300 K with the perpendicular anisotropy of 1.2 × 10^7^ erg/cm^3^ [313].

The TMR ratio has then been improved by tailoring the interface using Co to be 40% at RT for the MTJ, consisting of Mn_0.62_Ga_0.38_ (30)/Mg (0.4)/MgO (1.8)/CoFeB (1.2) (thickness in nm) [315,316]. Similar efforts have been devoted on polycrystalline MTJ consisting of Mn_3_Ge/MgO/CoFeB, which shows a negative TMR ratio of – 35 and – 75% at 300 and 3 K, respectively, and low *RA* of 10 Ω·µm^2^ [317].

#### (ii) MgO insertion

By inserting Co_2_MnSi between Mn-Ga and MgO, the perpendicular anisotropy of the Mn-Ga layer can induce perpendicular anisotropy in the half-metallic Co_2_MnSi layer, which is expected to achieve a large TMR ratio. Experimentally, TMR ratios of 10% at RT and 65% at 10 K have been achieved [318], which is smaller than the Mn-Ga/MgO/Mn-Ga junctions, as above. Additionally, the Co_2_MnSi magnetisation is in tilted states during the reversal process, which causes the TMR curves to be not well defined.

Similar to the CoFeB/MgO/CoFeB systems, perpendicular anisotropy has been induced by attaching a MgO tunnel barrier. In a p-MTJ, consisting of Co_2_FeAl/MgO/Co_0.2_Fe_0.6_B_0.2_, a TMR ratio of 53% has been reported at RT [277]. By inserting a 0.1-nm-thick Fe (Co_0.5_Fe_0.5_) layer between the MgO and Co_0.2_Fe_0.6_B_0.2_ layers, the TMR ratio was significantly enhanced to 91% (82%), due to the improved interface. The corresponding *RA* is 1.31 × 10^5^ Ω·µm^2^. By further improving the MTJ quality, consisting of Co_2_FeAl (1.2)/MgO (1.8)/Fe (0.1)/CoFeB (1.3) (thickness in nm), it has been reported to show TMR = 132% and *RA* = 1 × 10^6^ Ω·µm^2^ at RT [319].

By substituting some of Mg atoms, a MgAl_2_O_4_ [320] and MgGa_2_O_4_ [321] barrier can be formed, of which lattice constant matches perfectly with the Heusler alloy films. These spinel barriers show their corresponding TMR ratios with respect to their thickness. The height of MgGa_2_O_4_ is found to be much lower than that of MgAl_2_O_4_. A Co_2_FeAl/MgAl_2_O_4_ epitaxial heterojunction with perpendicular anisotropy has been investigated for the understanding of the origin of the perpendicular magnetic anisotropy [322]. The strong anisotropy at the interface is attributed to (1) the reduced in-plane magnetocrystalline anisotropy due to the lattice-matching and (2) the promoted hybridization between Fe and O orbitals due to the Al re-distribution near the interface.

#### (iii) PMA seed layer

A perpendicularly magnetised seed layer has also been used to induce perpendicular anisotropy onto the Heusler alloy films. For example, MTJ stack with *L*1_0_-CoPt/Co_2_MnSi/MgO/FePt has been demonstrated [323], as similarly reported in a conventional CoFeB/MgO/CoFeB junctions.

### 6. Antiferromagnetic Heusler alloys

For an HDD read head and an MRAM cell, an AF layer has been traditionally used to pin one of the FM magnetisations to achieve a well-defined antiparallel magnetisation configuration. Additionally, AF spintronics has been widely studied based on spin polarisation induced by flowing an electrical current in an AF layer [324]. For these spintronic applications, an IrMn_3_ alloy has been predominantly used due to its corrosion resistance and robustness against device fabrication processes in nanometre-scale in both thickness and in-plane dimensions. However, due to the scarcity of Ir as a platinum group metal, the price of IrMn_3_ has risen over 10 times in the last decade [325].

For the development of a replacement for IrMn_3_, RT antiferromagnetism needs to be achieved. However, the majority of the AF materials have their Néel temperature *T*_N_ near or below RT. Some oxides (*e.g*., NiO) and sulphides (*e.g*., CuFeS_2_) have *T*_N_ > RT but they have very poor corrosion resistance and hence cannot be used in a device. Manganese alloys (*e.g*., NiMn and PtMn) and nitrides (*e.g*., MnN and MnSiN_2_) also have *T*_N_ > RT. However, NiMn has poor corrosion resistance and PtMn has very high crystallisation temperature, indicating that they cannot be used in a device either. Therefore, there is strong demand for a new AF Heusler alloy to be developed.

A list of major AF and compensated ferrimagnetic (CF) Heusler alloys is shown in Table 7. In Heusler alloys, half-Heusler alloys have low Néel temperatures in general. For example, CuMnSb [326], NdBiFe [327] and GdPdBi [328] have their *T*_N_ to be 55, 2.18 and 13 K, respectively. These are not suitable for Ir-Mn replacement in spintronic devices due to their low *T*_N_ < RT and due to the use of rare materials in the latter two alloys. Therefore, full-Heusler alloys have been focused for the development of AF films with their *T*_N_ > RT.

**Table 7.** List of major AF and CF Heusler alloys and their Néel temperatures (*T*_N_), Curie temperatures (*T*_C_), average blocking temperatures (<*T*_B_>), exchange biases (*H*_ex_) and their forms, bulk, epitaxial (epi.) or polycrystalline (poly.) films or calculations (calc.). Simulated results using molecular dynamics (MF) are also shown

Heusler alloys	*T*_N_ [K]	*T*_C_ [K]	<*T*_B_> [K]	*H*_ex_ [Oe]	Forms	Refs.
CuMnSb	2				Bulk	[326]
NdBiFe	18				Bulk	[327]
GdPbBi	13				Bulk	[328]
Pt_2_MnGa	350	–			Bulk	[329]
Ru_2_MnGe	300	–			Bulk	[330]
	320				Simulations	
		–	126	>81 (100 K)	Epi. Films	[331]
	365	–			Calc.	[332]
Ru_2_MnSi	313	–			Calc.	
	335				Simulations	
Ru_2_MnSn	296	–				
Ru_2_MnSb	195	–				
Ni_2_MnAl	313	–			Bulk (B2)	[333]
	–	375			Bulk (L2_1_)	
	245	–			Calc. (B2-I)	[334]
	350	–			Calc. (B2-II)	
	–	368			Calc. (L2_1_)	
	340 372	–			Calc. (B2-I) MF Calc.	[335]
	220 352	–			Calc. (B2-II) MF Calc.	
	285	–			Calc. (B2-II)	
	310	–		>55 (10 K)	Epi. Films	[336]
Mn_2_VAl	>600	–			Bulk	[337]
	>RT	–	~200	120 (4 K) 20 (RT)	Epi. Films	
	360 636	–			Calc. MF Calc.	
Mn_2_VSi		–	<100	34 (100 K)	Poly. Films	[338]
Mn_3_Ga	470	–			Bulk	[339]
	648	–	~400	1.5 k (RT)	Epi. Films	[340]
		–	235	430 (120 K)	Poly. Films	[341]
Mn_3.04_Ge_0.96_			390			[342]
						[343]
Mn_3_Ge						[344]
Mn_2.4_Pt_0.6_Ga	–	~90		33 k (2 K) 0 (90 K)	Bulk	[345]
Mn_1.8_FeGa	–	~350		12 k (2 K) ~300 (RT)		[351]
Mn_2_FeGa						[346]
Mn_2.5_Co_0.3_Ga_1.2_	–		>350	250 (RT)	Poly. films	
MnN	660	–	388	1475 (RT)	Poly. Films	[347]
	570	–			Calc.	
Fe_2_VAl					Calc.	[348]
					Poly. films	[349]
Cr_2_MnSb	342				Calc.	[169]

As listed in Table 7, Fe_2_VAl, where Y and Z elements can be substituted with any other elements as listed in Figure 5, has been predicted to have a tendency to form a spin-glass (form AF ordering in Fe_2.5_V_0.5_Al) [348,349]. Mn_2_VAl is analogous to Fe_2_VAl but replaces the Fe with the very high moment Mn. These alloys can be further engineered by substituting some of Mn atoms with the other high-moment atoms to form (Co,Mn)_2_VAl for instance, which is analogous to the other two families with the exception that the element denoted X is now replaced by a mixture of two high moment atoms. By replacing V with Mn, low-moment-based alloys, *e.g*., Ni_2_MnAl, are anticipated to have a high compensated moment. By utilising a heavier element as a base of the Heusler alloys, Ru_2_MnAl is expected to have the potential advantage of having both X and Y as the high moment atoms. These families are anticipated to exhibit AF ordering. For RT antiferromagnetism, the AF phase should be stabilised by introducing larger anisotropy and larger AF grain volume. Recently, perpendicularly magnetised FM has also been reported to be pinned by IrMn layer [350]. Hence, the introduction of the additional tetragonal distortion into the cubic Heusler alloys may be necessary for the development of an RT AF.

### 6.1. Heavy-metal-based Heusler alloys

Epitaxially grown Ru_2_MnGe films have a very small lattice mismatch of 0.5% on a MgO(001) substrate with the relationship, Ru2MnGe[100](001)||MgO[110](001) (*a*_Ru2MnGe_ = 0.5985 nm, *c*_Ru2MnGe_ = 0.6041 nm and *a*_MgO_ = 0.5957 nm). At a substrate temperature *T*_sub_ > 673 K, the formation of epitaxial films has been reported [331] with the optimum growth temperature to be *T*_sub_ = 773 K for the *L*2_1_ phase formation. For an epitaxial Ru_2_MnGe/Fe bilayer. The mean blocking temperature <*T*_B_> is measured to be 126 K using the York protocol [351].

First principle calculations with advanced classical spin model simulations support the Ru-based bilayers to show exchange bias for the *L*2_1_ phase [352]. Due to the strong FM coupling between the interface atoms, the Fe and Mn spins are oriented nearly parallel in the vicinity of their interface, and the direction of these spins is in-plane following the in-plane magnetic anisotropy. The results confirm that the Ru-based Heusler alloys should be in the *L*2_1_ phase to exhibit AF behaviour. In order to increase the AF anisotropy and the resulting exchange bias *H*_ex_ induced in the neighbouring FM layer, calculations suggest that tetragonal distortion (stretching) can induce large uniaxial anisotropy in Ru_2_MnZ due to a combined effect of symmetry breaking and spin-orbit coupling. A similar AF behaviour has been reported for the other heavy-metal-based Heusler alloys, such as Pt_2_MnGa [340]. Therefore, for the heavy-metal-based Heusler alloys, high-moment element, *e.g*., Mn, is necessary to induce AF behaviour. The AF anisotropy can be increased by introducing tetragonal distortion, leading to RT AF behaviour.

### 6.2. Transition-metal-based Heusler alloys

Ni_2_MnAl films grown on MgO(001) have been reported to form the *L*2_1_ and *B*2 phase for 873 K and RT growth, respectively [337]. Only the latter phase exhibits the exchange bias onto a neighbouring FM layer with <*T*_B_> ≤100 K. Since the Ni_2_MnAl layer has a number of crystalline defects causing the random distribution of crystalline boundaries, the formation of a larger grain size may be required to increase thermal stability for RT antiferromagnetism.

Atomistic calculations confirm that no exchange bias occurs [353], however, a partially disordered *B*2 phase, a small *H*_ex_ has been found, which agrees with experiments as described above. Here, the disorder leads to uncompensated spin structures along the interface. Since the corresponding magnetic anisotropy is rather low (an order of magnitude smaller than that for the Ru-based Heusler alloys), *H*_ex_ is rather small and depends on the grain volume. It is furthermore rather unstable against thermal fluctuations with a maximum *T*_B_ of below 100 K as agreed with the experiment. Therefore, the Heusler alloys based with a transition metal with a small magnetic moment exhibits their AF phase when the high-moment Y atoms form antiparallel configuration between their second-nearest neighbours.

### 6.3 High-moment-metal-based Heusler alloys

As a Heusler alloy based with a transition metal with a high moment, Mn_2_VAl films have been grown onto MgO(001) single-crystalline substrates with forming the *L*2_1_ and *A*2 phase for *T*_sub_ ≥ 773 and ≤673 K, respectively. By depositing polycrystalline Mn_2_VAl/Fe bilayer, *H*_ex_ of 120 Oe at 10 K has been measured [341], corresponding to <*T*_B_> ~75 K.

Polarised neutron reflectivity (PNR) measurements confirmed the presence of an AF phase at RT in a polycrystalline *A*2-ordered Mn_2_VAl bulk sample. The Mn_2_VAl film deposited at 673 K is similarly found to show AF *A*2 phase at RT, while those deposited at RT and 873 K show the *A*2 phase without AF ordering and the *L*2_1_ phase, respectively [336].

The ordered Mn_2_VAl alloy has a FI ground state stabilised by a rather large V moment-oriented antiparallel to an Mn moment. According to atomistic calculations, the disorder between the V and Al atoms does not influence significantly the magnetic state as compared to the ordered *L*2_1_ state. The fully disordered *A*2 phase is also studied as a random three-component alloy in terms of single-site coherent potential approximation (CPA), showing NM. Only by increasing the lattice parameter by more than 8%, a spontaneous FM state has been formed. The failure of obtaining an AF ground state (in fact, a magnetic ground state) at the experimental lattice constant indicates that magnetism collapses when a homogeneous atomic disorder is supposed like with the CPA and, most possibly, atomic short-range order (*e.g*., clustering of Mn atoms) would stabilise the magnetic order in the system.

For the Mn-based Heusler alloys, off-stoichiometric compositions have also been investigated, which confirms the robustness of the Mn-based alloys against their atomic disorder. By taking two FI Heusler alloys, Mn_3_Ga and Mn_2_PtGa, their compensation point, Mn_2.4_Pt_0.6_Ga, has been calculated and demonstrated experimentally [345].

By further substituting Y elements with Mn, binary Heusler alloys can be formed. One example is hexagonal Mn_3_Ge [343]. *H*_ex_ of up to 520 Oe is measured at the boundaries between AF and FM domains. A tetragonal Mn_3_Ga film has then been investigated to induce AF behaviour [339]. A bilayer of epitaxial Mn_3_Ga (10 nm)/Co_0.9_Fe_0.1_ (2.5 nm) is reported to show *H*_ex_ of 1.5 kOe at RT. Magnetic anisotropy energy and <*T*_B_> are estimated to be 3 × 10^6^ erg/cm^3^ and ~400 K, respectively. Recently, *H*_ex_ of 430 Oe at 120 K in polycrystalline Mn_3_Ga/Co_0.6_Fe_0.4_ bilayers, confirming the applicability of such binary Heusler alloys for a device [341]. Similar off-stoichiometric AF Heusler alloys are reported as Fe_2_VAl [348,349] and Cr_2_MnSb [169].

By further expanding the definition of the Heusler alloys to nitrides [330], MnN has been investigated. MnN films are grown using ultrahigh vacuum sputtering in N_2_ atmosphere to achieve Mn:N = 1:1. A MnN/Fe bilayer has been reported to show *H*_ex_ of 1.4 kOe at RT with <*T*_B_> = 388 K [347]. However, the minimum thickness of MnN to induce the AF behaviour is 20 nm, which needs to be at least halved to be competitive against the 6-nm-thick Ir-Mn layer used in spintronic devices.

Therefore, high-moment-metal-based Heusler alloys display AF behaviour possibly due to the clustering of the high-moment metals even in their disordered *A*2 phase. Magnetic anisotropy is demonstrated to be increased by introducing tetragonal distortion into the unit cell of the alloys. Further engineering in distortion and AF domain size can increase <*T*_B_> and *H*_ex_ of these alloys, allowing the replacement for Ir-Mn alloys used in spintronic devices.

### 7. Major characterisations techniques of antiferromagnetic Heusler alloys

#### 7.1. Electrical resistivity

For the Néel temperature measurement, the temperature dependence of electrical resistivity has been utilised to determine *T*_N_ by detecting its gradient change [354]. Above *T*_N_, the moment alignment becomes random in the AF materials and changes the corresponding resistivity. As an example, a single-crystal of Cr with dimensions 5 × 5 × 1 mm^3^ has been measured to confirm the applicability of the resistivity measurements to determine *T*_N_ in a thin-film form [325]. A clear minimum of the order of µΩ is observed in the resistivity at 311 K. Measuring this change is only possible due to the low resistance of the crystal, *e.g*., 100-nm-thick epitaxial Ni_2_MnAl films grown at elevating temperature [337].

#### 7.2. X-ray magnetic linear dichroism

In order to characterise the AF materials microscopically, synchrotron radiation has been widely employed. X-ray magnetic linear dichroism (XMLD) utilises a pair of linearly polarised soft X-ray beams with perpendicular polarisation axes, which is different from a pair of circularly polarised beams used in XMCD as described in Sec. 4.4. Here, XMCD signals are proportional to the average value of a magnetic moment in a domain (<*M*>), while XMLD signals are proportional to <*M*^2^ > . For an AF material, <*M*> is zero as *M*_A_ = – *M*_B_ within an AF domain in Eq. (2), resulting in no XMCD signal. However, <*M*^2^> is a finite value for an AF material, allowing AF domain imaging. For example for an Fe/NiO bilayer, NiO domain structures have been observed by taking a Ni *L*-edge, which is strongly affected by the exchange coupling between Fe and NiO (spin image), and by taking the O *K*-edge, which is originated from the intrinsic AF anisotropy due to the strong coupling with the Ni 3*d* orbital (twin image) [355]. For these domain imaging, a large uniform domain (>a few µm) is required, which makes it difficult to be used for an AF thin films.

#### 7.3. Polarised neutron reflection

Polarised neutron reflectivity (PNR) is another synchrotron-based technique to determine magnetic properties of bulk and layered materials [356]. Due to the magnetic moment of neutron beam interacting with magnetic materials to be observed, not only layer structures, such as thickness, density, composition and interfacial roughness, but also in-plane magnetic moments can be measured. The former structural analysis is similar to X-ray reflectivity (XRR) measurements but with higher accuracy in a shorter scanning period (<1 min.). The latter magnetic information can be obtained by detecting the neutron reflection with its spins interacted with those in an AF and/or FM layers.

### 8. Applications of antiferromagnetic Heusler alloys

AF Heusler alloys are extremely attractive due to the absence of rare, expensive or toxic materials in their composition. Furthermore, they are fully compatible with other FM and NM Heusler alloys, with small lattice mismatches, large conduction band overlap and similar interfacial properties. Therefore, AF Heusler alloys are suitable for a wide range of applications, both for AF spintronics or as pinning layers in traditional spintronic devices.

A recent work by Nayak et al. has shown experimentally the existence of the anti-skyrmion in a tetragonal Heusler alloy Mn_1.4_Pt_0.9_Pd_0.1_Sn with the *D*_2d_ symmetry [357]. Lorentz TEM was used to observe the formation of this new class of skyrmion, which is an alternating form of the previously observed Bloch and Néel skyrmions. These structures are stable at above RT in the presence of a small field (0.2 T) and can be meta-stabilised at lower temperatures in the absence of an applied field.

AF Heusler alloys have attracted great attention in the storage industry as a viable material for read/write heads and for ultra-low damping MRAM. All-Heusler GMR devices have been realised [358], these are yet to reach the necessary MR characteristics and Heusler alloy devices with non-Heusler alloy spacer layers require conditions which are not back-end-of-line (BEOL) compatible. However, many magnetoresistive properties could potentially be realised in an all-Heusler exchange-biased GMR/TMR device. The potential for half-metallic antiferromagnets [359–361] which will have lattice and energy-band matching to the rest of the device stack mean that AF Heusler alloys are an exciting avenue for device applications.

AF spintronics is one of the key avenues of investigation for the improvement of spintronic devices [362,363]: Increases in device density due to zero stray fields, radiation hardness against magnetic interference and ultra-fast magnetisation dynamics. These advantages indicate that AF spintronics can exceed the potential of conventional spintronics. Heusler alloys provide an exciting avenue within this new area due to their desirable properties. More specifically, it is the compensated ferromagnetic Heusler alloys, such as Mn_3_Ga, which are of key interest [345,364].

The *D*0_22_ tetragonal Heusler alloys can be tuned compositionally to be fully compensated. The advantages of this are that the dipole field is zero even at the atomic scale. Furthermore, with the tetragonal Heusler alloys the high *T*_C_ -500 K means that the magnetic order is thermally robust. With Mn-Fe-Ga alloys exchange bias fields of greater than 3 T have been established, further paving the way for application in both ferro- and antiferromagnetic spintronics.

### 9. Non-magnetic Heusler alloys

#### 9.1. Topological insulators

Topological insulators are one of the Dirac materials which have a topological nontrivial band structure leading to unique quantum phenomena [365]. For example, Qi et al. have studied on a topological insulator β-Bi_4_I_4_ by applying high pressure in order to obtain the quasi-one-dimension [366]. Hybrid functional method has been used to calculate the electronic properties to prevent the underestimated band gap within the local density approximation or generalised gradient approximation. The simulations show a weak interaction between the along the AГYM path and strong dispersion along the BГ direction indicates a strong interaction within the chain. Therefore, quasi-1D characteristics of β-Bi_4_I_4_ are confirmed. Density functional theory calculations show that electronic instability occurs at a critical pressure of 11.5 GPa. An experimental study on resistivity as a function of pressure shows a direct relationship between the resistivity and the bandgap stage (open or close). The resistivity decreases rapidly above the critical pressure and superconductivity is observed in β-Bi_4_I_4_.

A topological surface state has been reported in LuPtBi and YPtBi [369]. Angle-resolved photoemission spectroscopy (ARPES) measurements confirmed the formation of a topological phase with a clear Dirac point at the Г position, which has been supported by *ab initio* calculations. Similar properties have been reported in ZrIrZ (Z = As, Sb or Bi) [368,369]. Experimental study and theoretical calculation have been carried out to investigate the structural and magnetotransport properties of LuNiBi single crystals [370]. Further investigation on a topological state in Heusler alloys may be required for the search for a robust topological insulator.

### 9.2. Spin gap-less semiconductors

**Figure 13.**
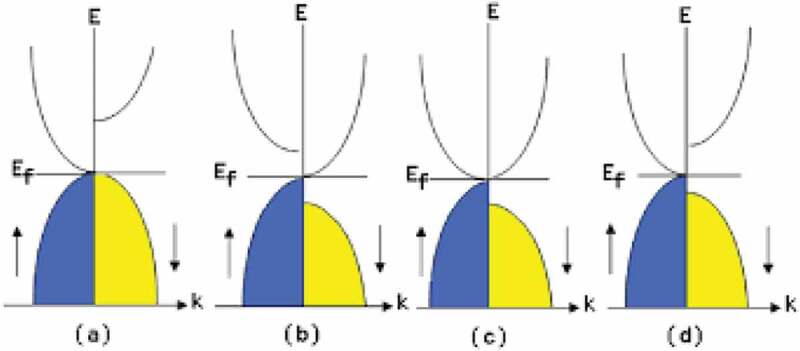
Four possible energy band structures for spin gapless semiconductors with parabolic dispersion energy against momentum [382]

Some Heusler alloys have been studied due to the unique property such as spin-gapless semiconductor (SGS). This novel material was first proposal by Wang et al. [371]. There are four possible band structures with spin gapless features as shown in Figure 13. These Heusler alloys have potential applications in spintronic devices because of their unique transport properties. For instance, almost no threshold energy is required to excite electrons from the valence to conduction band, the excited electrons have spin polarisation of 100%. Several SGS Heusler alloys such as Fe_2_CoSi [372], Mn_2_CoAl [373–374] CoFeCrGa [375] and Zr_2_MnGa [376] have been studied.

More than 10 FeRu-based quaternary Heusler alloys have been studied by Guo et al. [378–380]. FeRuCrSi is predicted to be a spin gap-less semiconductor with 100% spin polarisation. The total magnetic moment is calculated to be 2.0 μ_B_ [377]. Type-II ScMnVGa is a spin gap-less semiconductor in Mn-based Heusler alloys. The absence of a magnetic moment is calculated for ScMnVGa, which represents antiferromagnetic behaviour. This calculation is achieved using first principles calculations based on density functional theory [378] .

Xu et al. conducted detailed analysis on the magnetic properties of stoichiometric Mn_2_CoAl Heusler alloys. Saturation magnetisation at 5 K of the annealed stoichiometric and Co-rich Mn_2_CoAl samples are measured to be 2.6 and 2.5 μ_B_/f.u. respectively [379]. Bainsla et al. studied the magnetic behaviour of CoFeCrGa with high Curie Temperature of 620 K [380]. The saturation magnetisation of ≈ 2.1 μ_B_/f.u. is measured under zero applied pressure [375]. This value was in good agreement of *M*_s_ = 2.0 μ_B_ based on the generalised Slater-Pauling rule [65]. Fu et al. also studied magnetic properties of CoFeMnSi [381], as it has been predicted as a half-metallic ferromagnetic material [383]. The saturation magnetisation and coercivity are measured to be 98.6 emu/g (= 3.49 μ_B_/f.u.) and less than 25 Oe, respectively. The SGS Heusler alloys show ferromagnetic behaviour and exhibit soft magnetic behaviour.

**Figure 14.**
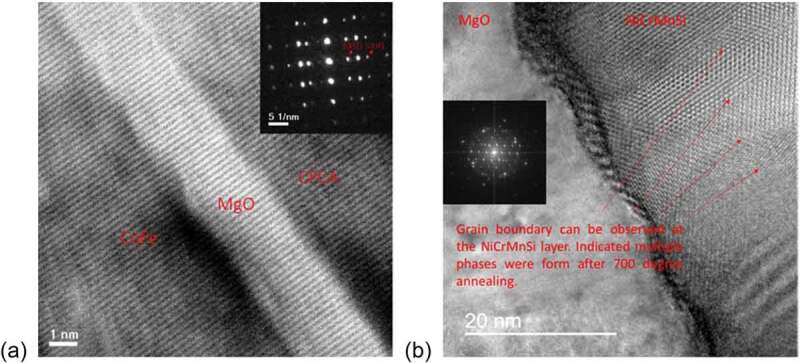
Cross-sectional TEM image of MTJ consisting of CoFeCrAl/MgO/CoFe and NiCrMnSi annealed at 973 K. Nanobeam diffraction patterns are also taken to confirm the crystalline ordering. Samples were grown by Mizukami et al. See Ref. [384] for details

These SGS Heusler alloy films have been used as a ferromagnetic layer in MTJs. For example, CoFeCrAl acts as a ferromagnetic layer in MTJ, showing 87% TMR ratio at RT [384]. Thin film sample is grown using magnetron sputtering technique, followed by post-annealing at *T*_anneal_ = 1,073 K. Cross-sectional TEM is performed to characterise the CoFeCrAl structure. Nanobeam diffraction confirms the chemical ordering of CoFeCrAl as shown in Figure 14(a) with very few interfacial dislocations. As discussed in Section 5.4., the TMR ratio of MTJ depends on various factors at the interface between ferromagnetic and insulator layers, such as the interface roughness and lattice mismatch [12]. The smooth interface induces a reasonably large TMR ratio. The diffraction spots of CoFeCrAl(002) and (004) are obtained, indicating that the CoFeCrAl exhibits the *B*2 (instead of *L*2_1_) ordering which agreed with the XRD data [384].

As another example, NiCrMnSi is used to examine the change of crystallinity using different annealing temperatures. Figure 14(b) shows cross-sectional TEM and the corresponding fast Fourier transformation (FFT) images NiCrMnSi is deposited on a MgO(001) substrate, followed by *in-situ* annealing at *T*_sub_ = 973 K. Diffraction pattern is also observed as an inset in Figure 14(b), indicating NiCrMnSi crystallisation.

## 10. Conclusion and future perspectives

The structural and magnetic properties of Heusler alloy films are explained from the viewpoint of spintronic applications. The crystallographical manipulation as well as spin-structural controllability can be achieved in the films by constituent element substitution. Recent progress in the film growth techniques enables the formation of almost perfectly ordered Heusler alloys. Local atomic disordering in the vicinity of theirs interface and surfaces prevents to achieve half-metallic electron transport at RT, which has been theoretically predicted for a bulk Heusler alloy. In order to achieve such surface asymmetry, first principles calculations and materials informatics using available databases [385],[386] have been employed to seek relevant combinations of interfacial atomic bonding, a surface termination and a crystalline orientation. Such an atomically controlled Heusler film is highly required for the use as a spin source for future spintronic devices.
